# Polymorphism, Weak Interactions and Phase Transitions in Chalcogen–Phosphorus Heterocycles

**DOI:** 10.1002/chem.201800978

**Published:** 2018-06-28

**Authors:** Paula Sanz Camacho, Martin W. Stanford, David McKay, Daniel M. Dawson, Kasun S. Athukorala Arachchige, David B. Cordes, Alexandra M. Z. Slawin, J. Derek Woollins, Sharon E. Ashbrook

**Affiliations:** ^1^ School of Chemistry and EaStCHEM and Centre of Magnetic Resonance University of St Andrews St Andrews, Fife KY16 9ST UK

**Keywords:** chalcogens, NMR spectroscopy, phosphorus heterocycles, polymorphism, X-ray diffraction

## Abstract

A series of P−E‐containing heterocycles (E=chalcogen) with aromatic backbones were synthesised and characterised by single‐crystal and powder XRD, microanalysis and mass spectrometry. Solution‐ and solid‐state ^31^P and ^77^Se NMR spectroscopy revealed significant differences between the NMR parameters in solution and in the solid state, related to conformational changes in the molecules. Many compounds were shown to exhibit a number of different polymorphic structures (identified by single‐crystal XRD), although the bulk material studied by solid‐state NMR spectroscopy often contained just one major polymorph. For the unoxidised heterocycles, the presence of weak intermolecular *J* couplings was also investigated by DFT calculations.

## Introduction

The study of polymorphism is of considerable importance, particularly where being able to tailor the properties of a given compound (e.g., solubility in the case of pharmaceuticals) is relevant for its final use.^1^ Although X‐ray crystallography is a useful tool for studying polymorphism,^2^, ^3^ solid‐state NMR spectroscopy also has a significant role to play in this area. NMR spectroscopy offers an element‐specific probe of the bulk material, providing direct information on the number of distinct species, and on any disorder and dynamics present.^4^, ^5^ In this respect, it can act as a bridge between the solution‐state NMR spectroscopic and crystallographic approaches that are widely used for the characterisation of molecular solids. Moreover, the dependence of the NMR parameters on molecular conformations and, importantly, on intermolecular interactions, provides a sensitive probe of the local environment and a convenient approach for distinguishing between polymorphs. These advantages have been exploited in recent years for the study of pharmaceutical polymorphs.^5^, ^6^ In principle, NMR spectroscopy of solids can also access more information than its solution‐state counterpart, as the anisotropic components of the NMR interactions (averaged by rapid tumbling in solution) also contain information on the local structure. For example, Wasylishen and co‐workers demonstrated that the ^77^Se chemical shift anisotropy (CSA) of square‐planar transition metal complexes of [N(*i*Pr_2_PSe)_2_]^−^ is very sensitive to changes in the conformation around the selenium centres.^7^ Furthermore, ^13^C and ^15^N CSAs have been used to characterise conformational polymorphs (i.e., a subclass of polymorphism, in which a molecule can adopt different conformations in the solid state through a controlled crystallisation process).^8^ The study of polymorphism by solid‐state NMR spectroscopy is often combined with periodic DFT calculations, to aid spectral assignment and interpretation, to calculate the relative energies of different polymorphs and to predict the most favourable structures.^9^, ^10^, ^11^, ^12^, ^13^, ^14^, ^15^ The ultimate goal in this area is to control the formation of a specific polymorph, but this can only be achieved if the thermodynamics and kinetics of the system are well known. In order to do this, all possible polymorphs and phase transitions as well as their thermodynamic stability and the kinetics of the phase transition must be known, and this requires the use of different (and complementary) techniques to address all of these complex questions.^16^

In 2015, Sanz Camacho et al.^17^ established the presence of extremely unusual through‐space interactions between Se and P atoms of adjacent molecules in naphthalene (Nap)‐based systems. This intermolecular *J* coupling was shown to be present for two compounds (**5** and **13** in this work), but only resolved in the ^77^Se spectrum of **13**. The *J* values calculated by periodic DFT confirm that a larger interaction is expected for **13**, as a consequence of the different packing motifs of the two compounds. To understand the effect of these unusual interactions on the stability, conformation and solid‐state packing of the compounds, the series has been extended here, both to include a different chalcogen (S) and to vary the oxidation state of the P atom, potentially precluding this atom's participating in additional interactions. A similar approach was taken in previous work by Woollins and co‐workers to monitor the resulting molecular distortion and effect on the though‐space interactions between the *peri* positions for compounds of the form Nap[P(E′)(Ph_2_)(ER)] (E′=O, S, Se).^18^, ^19^

Herein, we present a study on the properties and structural features of a series of new chalcogen–phosphorus heterocycles. These compounds exhibit extensive polymorphism, which was investigated not only by single‐crystal XRD, but also by studying the bulk material by solid‐state NMR spectroscopy and powder XRD (PXRD). Structural characterisation was completed by using solution‐state NMR spectroscopy, IR spectroscopy, mass spectrometry and elemental analysis. The isotropic chemical shifts *δ*_iso_ for ^77^Se and ^31^P were compared for solution and solid‐state samples, and differences were related to conformational changes.

## Results and Discussion

### Synthetic aspects

**Scheme 1:**
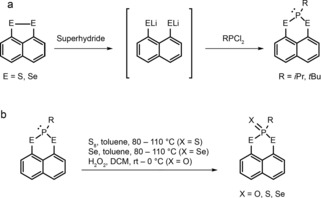
a) Preparation of **1**, **5**, **9** and **13** from naphtho[1,8‐*cd*]1,2‐dithiole or naphtho[1,8‐*cd*]1,2‐diselenole. b) Preparation of **2**–**4**, **6**–**8**, **10**–**12** and **14**–**16**. See Table 1 for numbering.

Scheme 1 shows the synthetic route for the preparation of the 16 organochalcogen heterocycles studied. Unoxidised heterocycles were prepared as shown in Scheme 1 a. Naphtho[1,8‐*cd*]1,2‐dithiole isopropylphosphine (**1**) and naphtho[1,8‐*cd*]1,2‐dithiole *tert*‐butylphosphine (**9**) were prepared according to reference 20. Naphtho[1,8‐*cd*]1,2‐diselenole isopropylphosphine (**5**) and naphtho[1,8‐*cd*]1,2‐diselenole *tert*‐butylphosphine (**13**) have already been described in recent work.^17^ The oxidised compounds were prepared as shown in Scheme 1 b, by using the procedure previously reported by Karacar et al.^21^, ^22^ (for 1,8‐bis(diphenylphosphino)naphthalene) for the sulfur and selenium analogues. The corresponding oxygen compounds were obtained by reaction with an excess of H_2_O_2_ at room temperature or 0 °C in air. Yields and compound numbering are given in Table 1.

**Table 1:** Organochalcogen heterocycles and their synthetic yields.

Compound	E, R, X	Yield [%]	Compound	E, R, X	Yield [%]
**1**	S, *i*Pr, –	66	**9**	S, *t*Bu, –	72
**2**	S, *i*Pr, O	93	**10**	S, *t*Bu, O	93
**3**	S, *i*Pr, S	56	**11**	S, *t*Bu, S	43
**4**	S, *i*Pr, Se	93	**12**	S, *t*Bu, Se	97
**5**	Se, *i*Pr, –	45	**13**	Se, *t*Bu, –	48
**6**	Se, *i*Pr, O	60	**14**	Se, *t*Bu, O	88
**7**	Se, *i*Pr, S	61	**15**	Se, *t*Bu, S	69
**8**	Se, *i*Pr, Se	77	**16**	Se, *t*Bu, Se	78

The P^III^ compounds **1**, **5**, **9** and **13** are stable in solution for up to about one week, after which decomposition to the NapE_2_ precursor is observed, rather than oxidation to the P^V^=O‐containing system. In the solid state, these compounds are stable upon exposure to air for at least 12 months. The oxidised heterocycles have high stability upon exposure to air in the solid form, but decompose quickly in solution to the E−E analogue (rather than the corresponding unoxidised heterocycle).

The single‐crystal X‐ray structures of **1**–**16** show some interesting features (see Table S1 in the Supporting Information). Six of the compounds (**1**, **3**, **4**, **8**, **12** and **16**) adopt different polymorphic forms (labelled a, b, c, etc.). There are a number of isomorphous structures (i.e., **2** and **6**; **3 b** and **8 a**; **3 c**, **7** and **8 b**; **11** and **12 b**; **15** and **16 a**). The basic molecular geometry is an open envelope conformation for the C_3_E_2_P ring with hinge angles between the C_3_E_2_ and E_2_P planes of about 55°. The molecules adopt two conformations. The substituent(s) on phosphorus (i.e., lone pair/X or R group) can be approximately perpendicular or collinear with the naphthalene plane. This can be thought of as similar to the axial/equatorial arrangements in a cyclohexane ring. The most obvious common feature is that all of the structures where X=O have the P=O bond approximately perpendicular to the naphthalene ring plane. Interestingly, in most cases (except for **15, 16 a** and **16 b**), for the heavier chalcogens, when R=*i*Pr the structures have the P−C bond perpendicular to the naphthalene plane, whereas the *t*Bu analogues have the P−C bond collinear.

### Solution‐state NMR spectroscopy

The ^31^P and ^77^Se NMR parameters for all compounds are given in Table 2. The ^31^P{^1^H} NMR spectra of all the complexes exhibit singlets with satellites due to ^1^ *J*(P−Se) or/and ^1^ *J*(P=Se) coupling for the selenium compounds. The ^31^P{^1^H} NMR spectra of the sulfur compounds appear to be the most deshielded of the series, in agreement with previous literature.^13^ In addition, the *t*Bu compounds are deshielded in comparison to the corresponding *i*Pr compounds, with the exception of **10**. The nature of the X substituent has an influence on the ^1^ *J*(^31^P−^77^Se) coupling constant, which decreases in the order O≥Se>S. The R group also has an impact on the ^1^ *J*(^31^P−^77^Se) coupling, with *t*Bu>*i*Pr. However, the opposite trend is observed for the ^1^ *J*(^31^P=^77^Se) coupling, which decreases in the order *i*Pr>*t*Bu. The ^77^Se isotropic chemical shifts are also affected by the nature of X, with *δ*_iso_(^77^Se) decreasing for the *i*Pr compounds in the order Se≥S>O. However, for the *t*Bu compounds, the S‐containing system is the most deshielded. In contrast to the ^31^P{^1^H} NMR spectra, *δ*_iso_(^77^Se) are, as expected, more deshielded for the *i*Pr analogues.

**Table 2:** Solution‐state (CDCl_3_, 6.35 T) NMR parameters (^31^P and ^77^Se isotropic chemical shifts *δ*_iso_ and ^31^P–^77^Se *J* couplings).

	**1**	**2**	**3**	**4**	**5**	**6**	**7**	**8**
E, R, X group	S, *i*Pr, ‐	S, *i*Pr, O	S, *i*Pr, S	S, *i*Pr, Se	Se, *i*Pr, –	Se, *i*Pr, O	Se, *i*Pr, S	Se, *i*Pr, Se
*δ*_iso_(^31^P) [ppm]	4.7	52.0	67.8	52.3	−3.4	40.4	43.3	22.0
*δ*_iso_(^77^Se) (ppm)	–	–	–	−310.6	270.2	403.8	438.7	439.2 −260.0^[a]^
*J*(^31^P–^77^Se) [Hz]	–	–	–	797	276	397	385	391 773^[b]^

	**9**	**10**	**11**	**12**	**13**	**14**	**15**	**16**
E, R, X group	S, *t*Bu, ‐	S, *t*Bu, O	S, *t*Bu, S	S, *t*Bu, Se	Se, *t*Bu, –	Se, *t*Bu, O	Se, *t*Bu, S	Se, *t*Bu, Se
*δ*_iso_(^31^P) [ppm]	24.1	51.1	70.2	53.8	12.3	44.1	48.6	27.3
*δ*_iso_(^77^Se) (ppm)	–	–	–	−152.5	210.2	392.3	413.2	406.1 −143.7[a]
*J*(^31^P–^77^Se)/Hz	–	–	–	790	302	407	398	407 752[b]

[a] P=Se. [b] ^1^ *J*(^31^P=^77^Se).

### Solid‐state characterisation of P^III^ heterocycles

**Figure 1:**
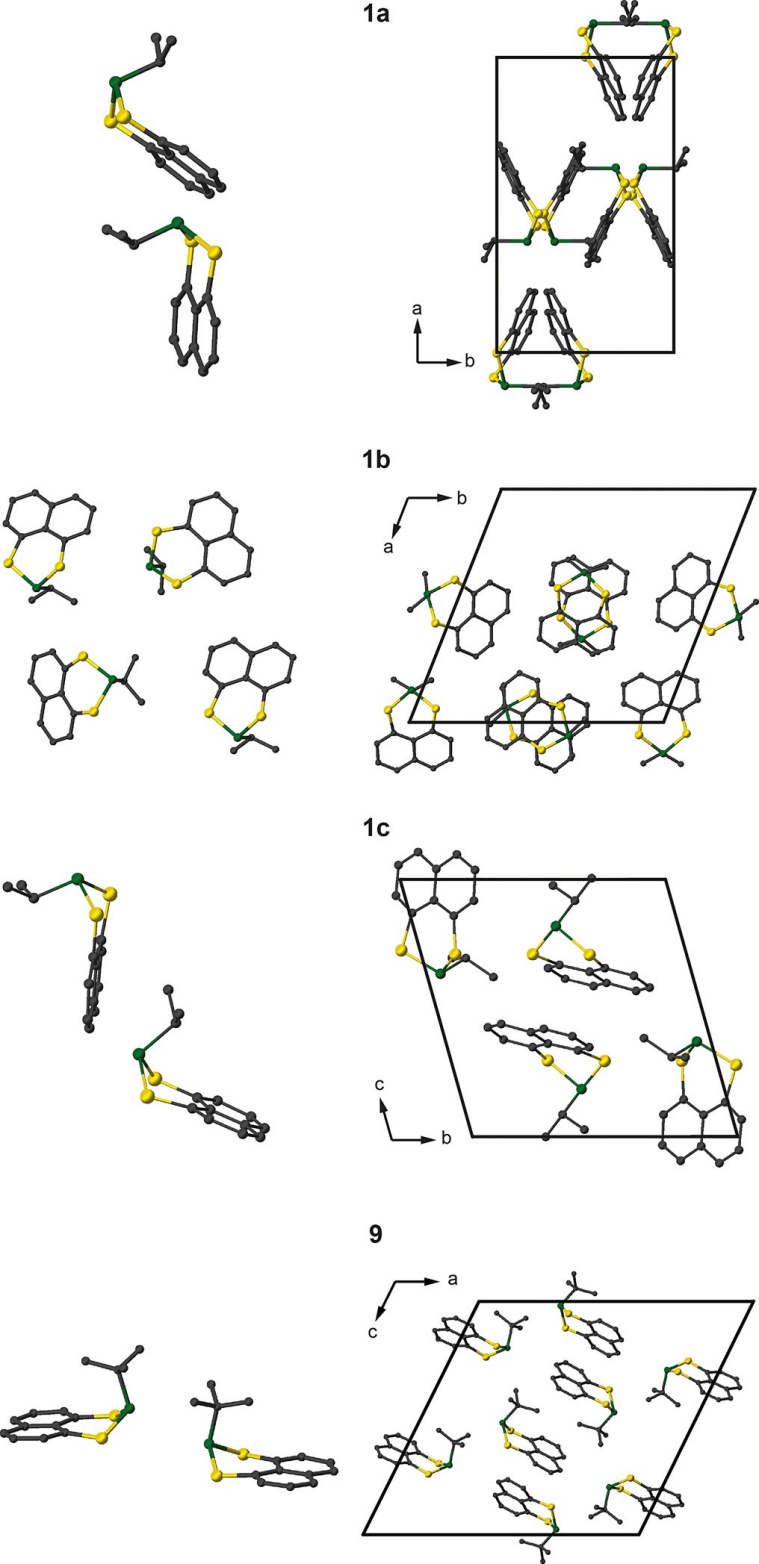
Asymmetric units and crystal packing for the three polymorphs of **1** and the single polymorph of **9**. Atoms are coloured with C=grey, P=green, S=yellow. Hydrogen atoms are omitted for clarity.

Compounds **5** and **13** have previously been characterised and studied by ^31^P and ^77^Se solid‐state NMR spectroscopy. The presence of an intermolecular *J* coupling between ^31^P in one molecule and ^77^Se in an adjacent molecule was observed for **13** (the *t*Bu analogue).^17^ This was not resolved in the ^77^Se NMR spectrum of **5** (where R=*i*Pr), as the different packing motifs of the two result in a greater distance between the two atoms and, therefore, a smaller coupling. In contrast, the sulfur compounds (**1** and **9**) have not been previously reported. Compound **1** exhibits three different polymorphs. The asymmetric units and packing motifs for these three structures and the single polymorph observed for **9** are shown in Figure 1. The three polymorphs of **1** differ not only in their asymmetric units, but also in the packing motifs. Polymorph **1 b** has four molecules in the asymmetric unit. Each molecule is more isolated and stacks in an antiparallel arrangement along the *c* axis. Both **1 a** and **1 c** have only two molecules in the asymmetric unit, although the extended packing is different between the two polymorphs. In **1 a**, the molecules form triangles that stack along the *c* axis, whilst in **1 c** the chains of molecules stack perpendicularly along the *a* axis. To determine which polymorph(s) were present in the bulk sample, a PXRD pattern was collected and compared to those simulated for each polymorph individually. The experimental and simulated PXRD patterns are shown in Figure S2.1 of the Supporting Information. The relative intensities and position of the reflections in the experimental pattern agree with those simulated for **1 b**.

The ^31^P MAS NMR spectra of **5** and **13** have previously been reported by Sanz Camacho et al., in 2016.^20^ In both cases, a single resonance is present with a significant sideband manifold, in agreement with the presence of a single molecule in the asymmetric unit. These authors also reported another unusual coupling in **13**, with a ^31^P *J*‐resolved spectrum showing an intermolecular homonuclear ^31^P–^31^P coupling (≈88 Hz). Interestingly, this interaction was between P species that are crystallographically equivalent but are rendered magnetically inequivalent in a fraction of the molecules by heteronuclear coupling to ^77^Se. This interaction was not resolved for **5** (although it was shown by DFT calculations to be present with a lower magnitude) as a consequence of the different packing motifs.

The ^31^P MAS NMR spectrum of **1**, shown in Figure S2.2 of the Supporting Information, contains three isotropic resonances, each with a significant sideband manifold as a result of CSA. The three resonances exhibit an integrated intensity ratio (including sidebands) of about 1:1:2, suggesting that the resonance at lowest shift might correspond to two P atoms with a very similar environment. This suggests the presence of four molecules in the asymmetric unit and hence the presence of polymorph **1 b** in the bulk sample, in agreement with the PXRD results. Unfortunately, due to the nature of the sample of **9** (a sticky solid), it was not possible to study the bulk compound by solid‐state NMR spectroscopy or PXRD.

**Figure 2:**
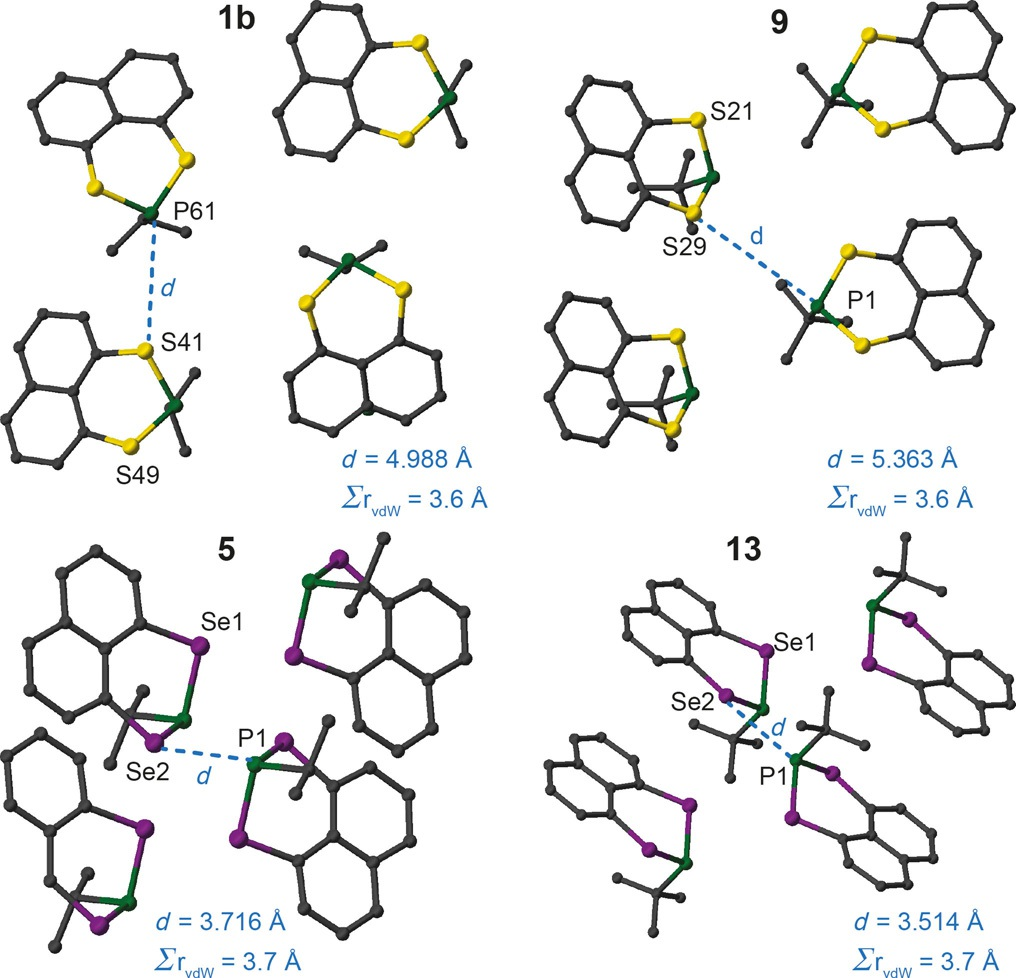
Crystal packing motifs for **1 b**, **5**, **9** and **13**, showing the shortest intermolecular Se−P and S−P distances and the sum of the van der Waals radii. Atoms are coloured with C=grey, P=green, S=yellow and Se=purple. H atoms are omitted for clarity.

Despite their chemical similarity, **1**, **5**, **9** and **13** exhibit different crystal packing motifs, resulting in different internuclear chalcogen–P distances, as shown in Figure 2. The two selenium compounds have much shorter contacts (within, or close to, the sum of the van der Waals radii) than the corresponding sulfur analogues. As discussed above, for **13** this leads to the observation of intermolecular (^31^P–^77^Se and ^31^P–^31^P) *J* couplings. Figure 2 suggests that similar ^31^P–^33^S couplings would not be present (even if the experimental challenges of ^33^S NMR spectroscopy could be overcome).

The intermolecular proximity in **13** also leads to the observation of ^31^P–^31^P homonuclear intermolecular coupling, as discussed above. Table 3 lists *J*_PP_ coupling constants predicted for compounds **1**, **5**, **9** and **13** by periodic DFT. Values of *J* are generally larger for shorter P−P distances, though, notably in **5**, a larger *J* value is predicted for the longer P−P distance owing to the relative orientation of the paired molecules. A significant through‐space *J* coupling is computed only for **13**, and this reflects the shorter internuclear P−P distance and particular packing arrangement found for this compound.

**Table 3:** Calculated homonuclear through‐space ^31^P–^31^P *J* couplings $J_{\mathrm{PP}}^{\mathrm{calcd}}$ , predicted by DFT at the scalar‐relativistic ZORA level of theory, and internuclear distance of the coupled P−P pair before (P−P^crystal^) and after (P−P^calcd^) optimisation.

Compound	$J_{\mathrm{PP}}^{\mathrm{calcd}}$ [Hz]	P−P^calcd^ [Å]	P−P^crystal^ [Å]
**1 a**	2^[a]^	5.400	5.494
**1 b**	0	6.391	6.454
**1 c**	11^[a]^	3.961	4.034
**5**	4^[a]^	4.814	4.901
**5**	11^[a]^	5.458	5.457
**9**	5^[b]^	5.914	6.349
**13**	159^[a]^	3.500	3.586

[a] 2×1×1 supercell. [b] 1×2×1 supercell.

### Solid‐state characterisation of oxidised (P=O) heterocycles

**Figure 3:**
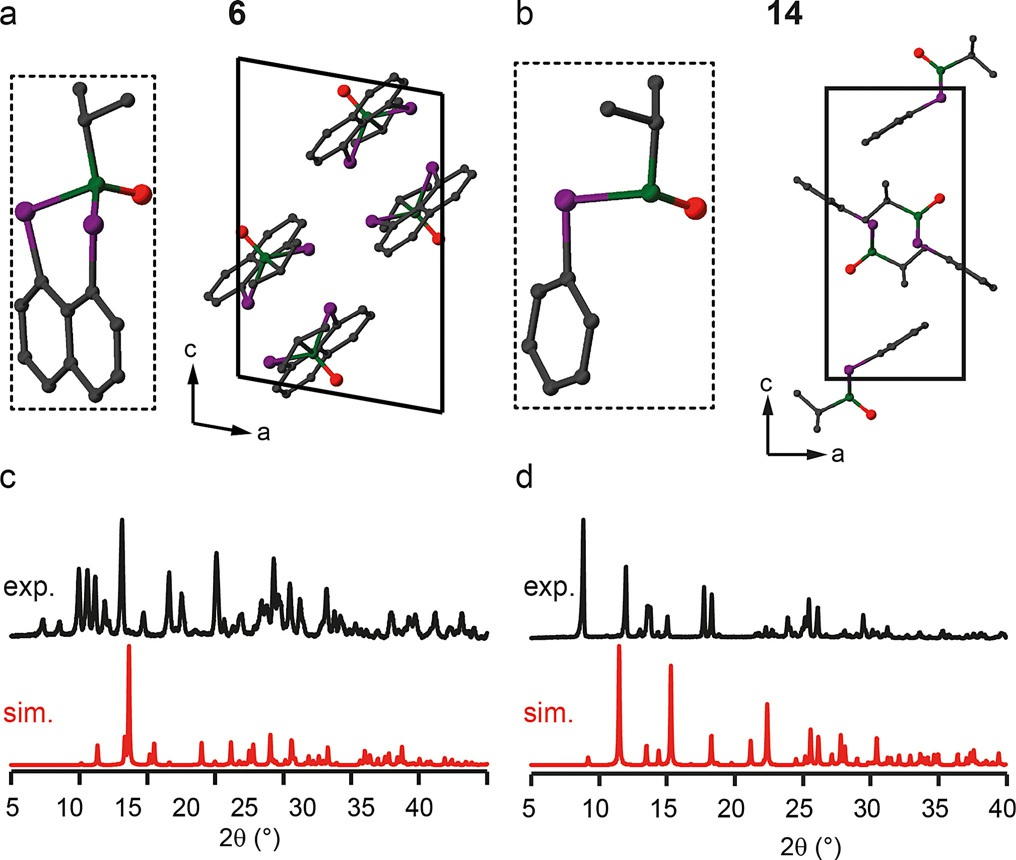
a, b) Asymmetric units (broken lines) and crystal packing, and c, d) comparison of the experimental and calculated PXRD patterns for **6** (a, c) and **14** (b, d). Atoms are coloured with C=grey, P=green, Se=purple and O=red. H atoms are omitted for clarity.

The four P=O oxidised heterocycles (**2**, **6**, **10** and **14**) were the most difficult to synthesise, due to their tendency to decompose, and only a single crystal structure was obtained for each compound. The asymmetric unit and packing motifs are shown in Figure 3 a and b for **6** and **14**, respectively, and in Figures S3.1 and S3.2 in the Supporting Information for **2** and **10**. The PXRD patterns for bulk samples of **2** and **10** (also shown in the Supporting Information) are in good agreement with those simulated from the structural models derived from single‐crystal diffraction. The ^31^P MAS NMR spectra (Supporting Information) also confirm the presence of one distinct P species. For selenium compounds **6** and **14**, the simulated PXRD patterns do not agree with those obtained experimentally for the bulk material, as shown in Figure 3 c and d, and this suggests that a different polymorph forms the majority of the bulk material. Repeated attempts to crystallise this polymorph were unsuccessful.

**Figure 4:**
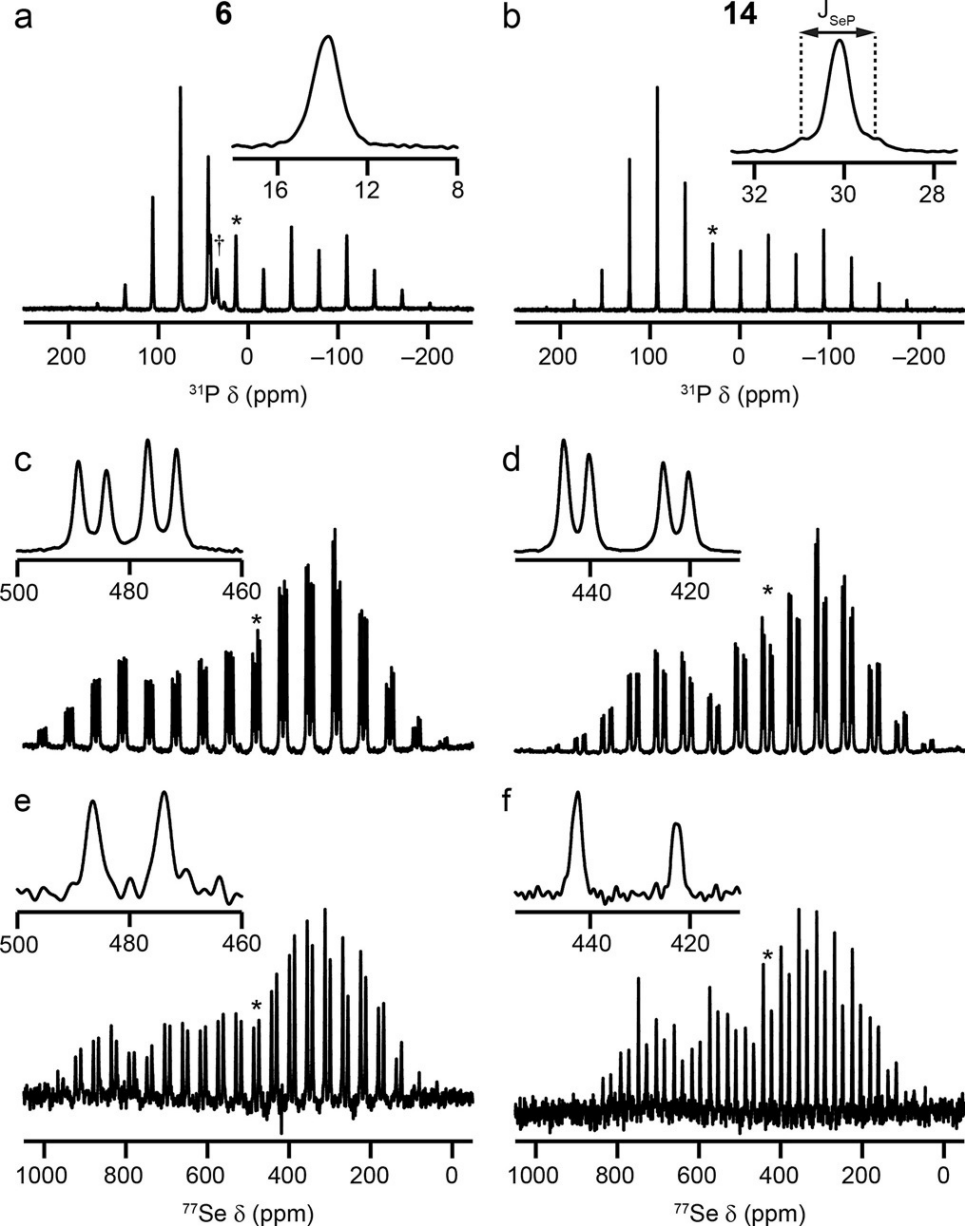
a, b) ^31^P (14.1 T, 7.5 kHz) MAS NMR spectra, c, d) ^77^Se (9.4 T, 5 kHz) CP MAS NMR spectra and e, f) ^77^Se (14.1 T, 5 kHz) CP MAS NMR spectra with ^31^P decoupling of **6** (a, c, e) and **14** (b, d, f). Isotropic resonances are marked with ✶ and expanded in insets. In a), resonances arising from decomposition of **6** are indicated with †.

Solid‐state ^31^P and ^77^Se NMR spectra of **6** and **14** are shown in Figure 4. The ^77^Se CP MAS NMR spectrum of **6** in Figure 4 c appears to show two doublets (with ^31^P–^77^Se couplings of 378 and 389 Hz). This can be confirmed by the application of ^31^P decoupling, as in Figure 4 e, after which two isotropic peaks are observed. In addition to the single isotropic peak (at ≈14 ppm) and its corresponding sideband manifold, a number of additional resonances (at ≈27, ≈35 and ≈42 ppm) are observed in the ^31^P MAS NMR spectrum (indicated with † in Figure 4 a), which are attributed to breakdown products. As shown in Tables 2 and 4), this isotropic shift is very different from that in solution (≈40 ppm). These data suggest that the major polymorph found in the bulk material has one molecule in the asymmetric unit (as is also seen for the polymorph found by single‐crystal diffraction), but that different crystal packing must be present given the different predicted PXRD pattern. For **14**, the polymorph found in the bulk material also has a single distinct ^31^P species and two distinct ^77^Se species (each with a single coupling to ^31^P), as seen in Figure 4 b, d and f. This is in contrast to the polymorph studied by single‐crystal XRD, which has only single distinct P and Se sites (Figure 3 b). As described above, it was not possible to grow single crystals of the polymorphs found in the bulk, despite repeated attempts. Although it is, in principle, possible to solve structures from PXRD data,^23^, ^24^ this is very challenging and was also not possible from the data we have for **14**. However, this clearly demonstrates the need for multiple characterisation techniques when synthesising new materials.

**Table 4:** Solid‐state NMR parameters (^31^P and ^77^Se isotropic chemical shifts *δ*_iso_ and ^31^P–^77^Se *J* couplings).

	**1**	**2**	**3**	**4**	**5**	**6**	**7**	**8**
E, R, X group	S, *i*Pr, –	S, *i*Pr, O	S, *i*Pr, S	S, *i*Pr, Se	Se, *i*Pr, ‐	Se, *i*Pr, O	Se, *i*Pr, S	Se, *i*Pr, Se
*δ*_iso_(^31^P) [ppm]	2.7	36	66	55	−2	14	41	26
	3.6		64				44	
	5.9		62					
*δ*_iso_(^77^Se) [ppm]	–	–	–	−309	280	487	441	409
						474	439	442
							432	−259^[a]^
							412	
*J*(^31^P–^77^Se) [Hz]	–	–	–	779	300	378 389	349 332 391 378	392 382 749^[b]^

	**9**	**10**	**11**	**12**	**13**	**14**	**15**	**16**
E, R, X group	S, *t*Bu, –	S, *t*Bu, O	S, *t*Bu, S	S, *t*Bu, Se	Se, *t*Bu, –	Se, *t*Bu, O	Se, *t*Bu, S	Se, *t*Bu, Se
*δ*_iso_(^31^P) [ppm]	–	47	72	54	6	30	43	21
			71	56				23
*δ*_iso_(^77^Se) [ppm]	–	–	–	−46	213	443	364	369
				−56	179	423	358	359
								356
								−151^[a]^
								−165^[a]^
*J*(^31^P–^77^Se) [Hz]	–	–	–	835	340/270^[c]^	384	396	411
				826	319	386	424	382
								347
								740^[b]^
								746^[b]^

[a] P=Se. [b] ^1^ *J*(^31^P=^77^Se). [c] One is an intermolecular through‐space *J* coupling.^17^

### Solid‐state characterisation of oxidised (P=S) heterocycles

**Figure 5:**
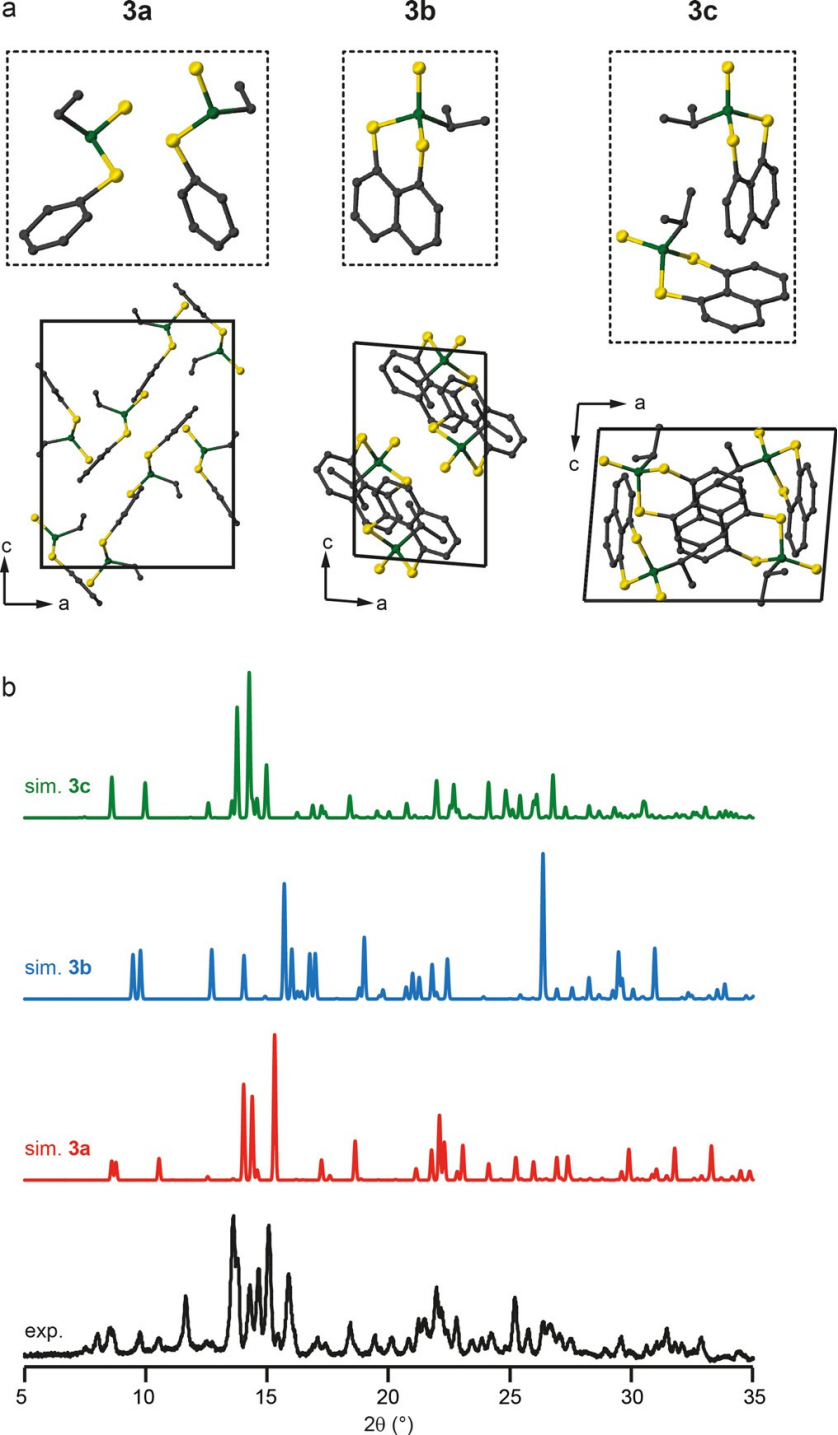
a) Asymmetric units (broken lines) and crystal packing for the three polymorphs of **3**. Atoms are coloured with C=grey, P=green and S=yellow. H atoms are omitted for clarity. b) Comparison of the experimental PXRD pattern of the bulk sample of **3** with those predicted for each polymorph.

**Figure 6:**
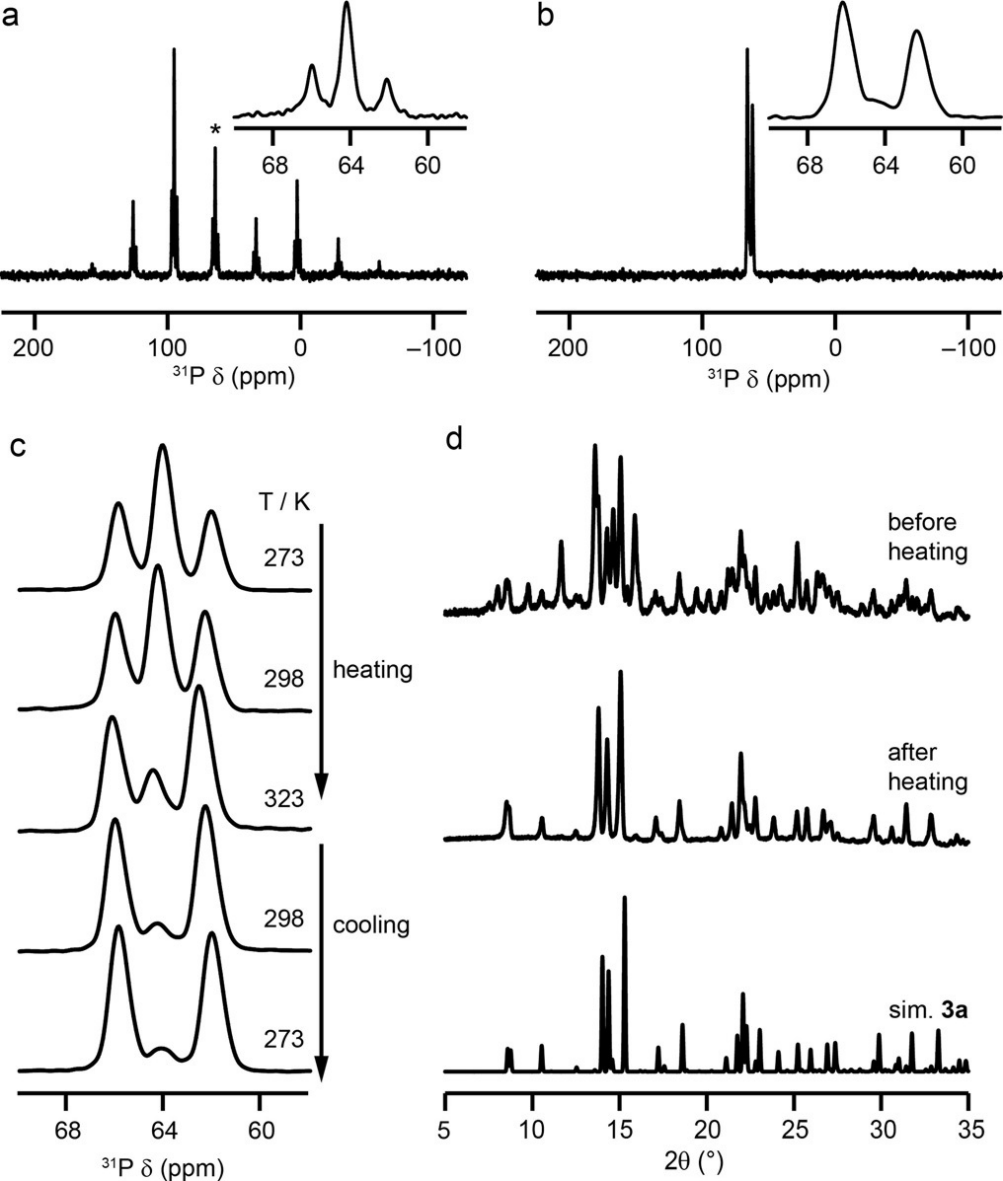
^31^P (14.1 T) NMR spectra of **3** acquired with a) 7.5 kHz and b) 55 kHz MAS for **3**. The inset shows an expansion of the isotropic region. c) ^31^P (14.1 T, 35 kHz) VT NMR spectra, with only the isotropic centre band shown for clarity. d) Comparison of the experimental powder XRD patterns of **3** before and after the VT NMR experiments and the pattern simulated for **3 a**.

The four P=S oxidised heterocycles are **3**, **7**, **11** and **15**. The asymmetric units, crystal packing motifs, PXRD data and ^31^P MAS spectra of **7**, **11** and **15** are shown in Section S4 of the Supporting Information. Only one polymorph is observed for each of **7**, **11** and **15**, with two, two and one distinct molecules in their respective asymmetric units. Their PXRD patterns are also in good agreement with those simulated from the structural models derived from single‐crystal XRD. For **3**, three different polymorphs are observed; **3 a** and **3 c** have two distinct molecules in the asymmetric unit, while only one is present for **3 b**. The difference in crystal packing motifs for the three polymorphs is shown in Figure 5 a. Simulated PXRD patterns for the three structures are very different, and comparison with the experimental powder XRD pattern for the bulk sample suggests it is a mixture of the three polymorphs, as shown in Figure 5 b. Unfortunately, it is not possible to determine the fractions of each polymorph in the bulk sample from these data. The ^31^P MAS NMR spectrum of **3** shows three resonances, each with different intensity, as shown in Figure 6 a. If all three polymorphs are present in the bulk material, as suggested by PXRD, five distinct resonances would be expected (two for each of **3 a** and **3 c** and one for **3 b**), although the chemical similarity of the environments may well result in some overlap of the spectral resonances.

The presence of significant sideband manifolds for each resonance hinders the accurate determination of relative signal intensities, and so a second spectrum was acquired at a faster MAS rate of 55 kHz (with a 1.3 mm rotor), as shown in Figure 6 b. A clear change is observed in the relative intensities of the isotropic peaks, with almost complete loss of the central signal. This is clearly not the result of averaging of the CSA, as this signal was the most intense in each of the spinning sidebands in Figure 6 a. Fast MAS, however, does result in an increase in the sample temperature (by ≈20 °C when spinning at 7.5 kHz MAS to ≈60 °C when spinning at 55 kHz MAS, without temperature regulation) as a result of frictional heating. It is possible that this change caused a phase transition and a change in the relative proportions of each polymorph present in the bulk sample. To investigate this further, variable‐temperature (VT) NMR experiments were performed for a different batch of the same compound. The resulting ^31^P MAS NMR spectra (isotropic region only) are shown in Figure 6 c. As the temperature is increased from 273 to 323 K, the central resonance was lost and the relative intensities of the remaining two peaks change. (There are also some small changes in chemical shift as the temperature varies.)

The changes appear to be irreversible, with no further change in the spectrum as the temperature is reduced back to 273 K. PXRD measurements also confirmed a change in the bulk sample after the VT NMR experiments, as shown in Figure 6 d. The pattern obtained after heating is in good agreement with that simulated for **3 a** (see also Figure S4.6 of the Supporting Information), which suggests loss of **3 b** and **3 c** from the bulk material. This suggests that the two resonances observed at about 62 and about 66 ppm correspond to the two distinct P species in **3 a**, while the signal at about 64 ppm most likely results from the overlap of three signals (from **3 b** and **3 c**).

### Solid‐state characterisation of P=Se oxidised heterocycles

Two polymorphs were identified for each of the P=Se oxidised compounds (**4**, **8**, **12** and **16**). The asymmetric units, crystal packing motifs and PXRD data for **4**, **8** and **16** are shown in Section S5 of the Supporting Information. The two polymorphs of **4** have one (**4 a**) and two (**4 b**) distinct molecules in the asymmetric unit and have very different predicted PXRD patterns. Comparison of these to the experimental PXRD pattern suggests that **4 a** makes up the majority of the bulk material. The ^31^P MAS and ^77^Se CP MAS NMR spectra (Figure S5.2 in the Supporting Information) exhibit one ^31^P and one ^77^Se species, in agreement with the structure of **4 a**, together with low‐intensity resonances that suggest **4 b** is present only in a very small amount. The ^1^ *J*(^31^P=^77^Se) coupling can be resolved in both the ^31^P and ^77^Se spectra (≈779 Hz). Upon application of ^31^P decoupling, the ^77^Se spectrum displays a very small residual coupling, most probably as a result of the low decoupling power that can be applied and the magnitude of the CSA present. The two polymorphs of **8** (Figure S5.3 in the Supporting Information) have one (**8 a**) and two (**8 b**) distinct molecules in the asymmetric unit, again with very different predicted PXRD patterns. Comparison with the experimental PXRD pattern suggests that the bulk material is primarily **8 a**. The ^31^P MAS and ^77^Se CP MAS NMR spectra (Figure S5.4 in the Supporting Information) show a single P site and three distinct Se sites, as expected, with ^1^ *J*(^31^P−^77^Se)=380–390 Hz and ^1^ *J*(^31^P=^77^Se)=749 Hz.

The two polymorphs of **16** have one (**16 a**) and two (**16 b**) molecules in their asymmetric units but have very similar predicted PXRD patterns (Figure S5.5 in the Supporting Information). The ^31^P MAS NMR spectrum (Figure S5.6 in the Supporting Information) contains two closely spaced resonances with similar intensities, suggesting that either 1) **16 b** makes up the majority of the bulk material, with **16 a** present only in small amounts, or 2) the two P atoms in **16 b** have an identical chemical shift and there is a 2:1 ratio of **16 a** and **16 b**. The ^77^Se CP MAS NMR spectra (also shown in Figure S5.6 of the Supporting Information) confirm that six Se sites are most likely to be present, again in support of the presence of only **16 b** in the bulk sample, although resonances overlap even under ^31^P decoupling. Final confirmation was obtained by acquiring the ^77^Se CP MAS NMR spectrum of a powder obtained from a single crystal whose structure was determined by single‐crystal XRD to be **16 b**. This was in excellent agreement with the spectra obtained on the bulk material.

**Figure 7:**
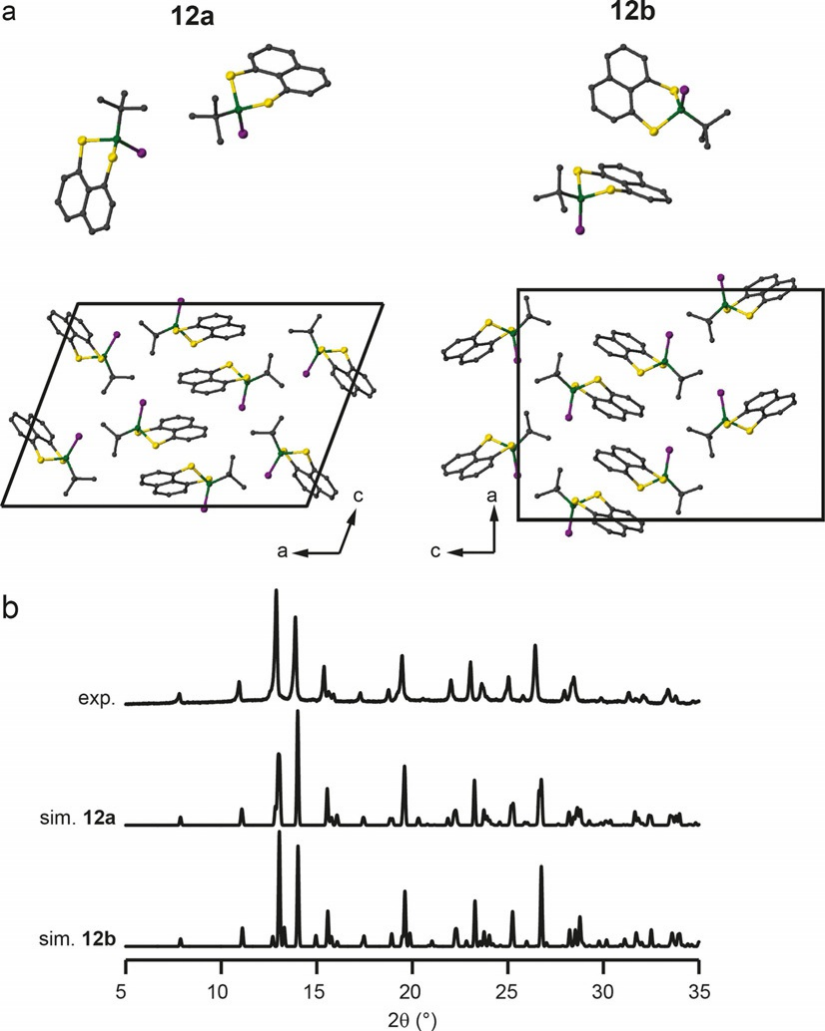
a) Asymmetric units and crystal packing and b) comparison of the experimental and calculated PXRD patterns for **12 a** and **12 b**. Atoms are coloured with C=grey, P=green, S=yellow and Se=purple. H atoms are omitted for clarity.

**Figure 8:**
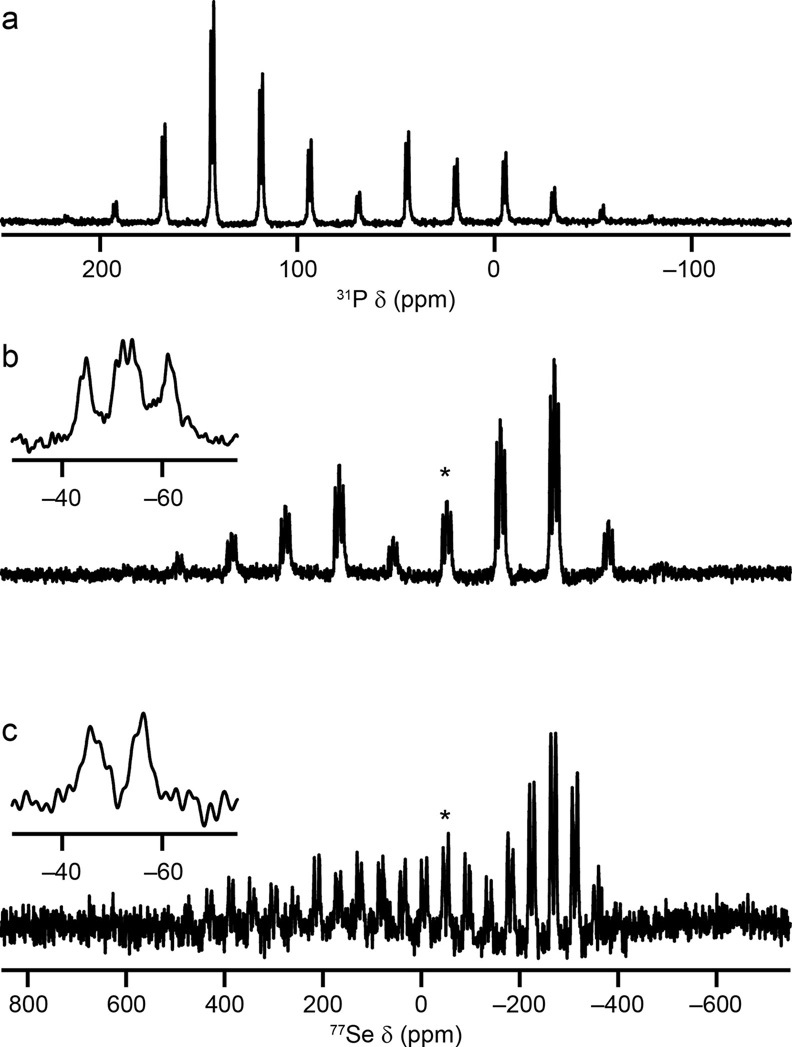
a) ^31^P (14.1 T, 7.5 kHz) MAS NMR spectrum of **12**. b, c) ^77^Se (9.4 T, 5 kHz) CP MAS NMR spectra of **12**, acquired b) without and c) with ^31^P decoupling. Isotropic resonances are marked with ✶ and expanded in insets.

Polymorphs **12 a** and **12 b** both contain two molecules in the asymmetric unit, although the two structures have different crystal packing motifs, as shown in Figure 7 a. Both polymorphs were produced from the same synthetic batch, but **12 a** resulted from crystallisation from dichloromethane/ethanol, while **12 b** crystallised from dichloromethane/methanol. The very similar simulated PXRD patterns (Figure 7 b) make it difficult to determine the fraction of each polymorph in the bulk sample. There are only two resonances in the ^31^P MAS NMR spectrum (Figure 8 a), possibly corresponding to two inequivalent sites in just one of the polymorphs. The ^77^Se CP MAS NMR spectrum (Figure 8 b) exhibits two doublets (with ^1^ *J*(^31^P=^77^Se) couplings of about 835 and about 826 Hz. Additional peaks are also observed as shoulders on each resonance, possibly arising from the second polymorph. The application of ^31^P decoupling suggests that four Se sites are present, although the low decoupling power available does limit resolution. From this, it seems likely that a mixture of both polymorphs is present in the bulk sample.

### Conformational differences

**Figure 9:**
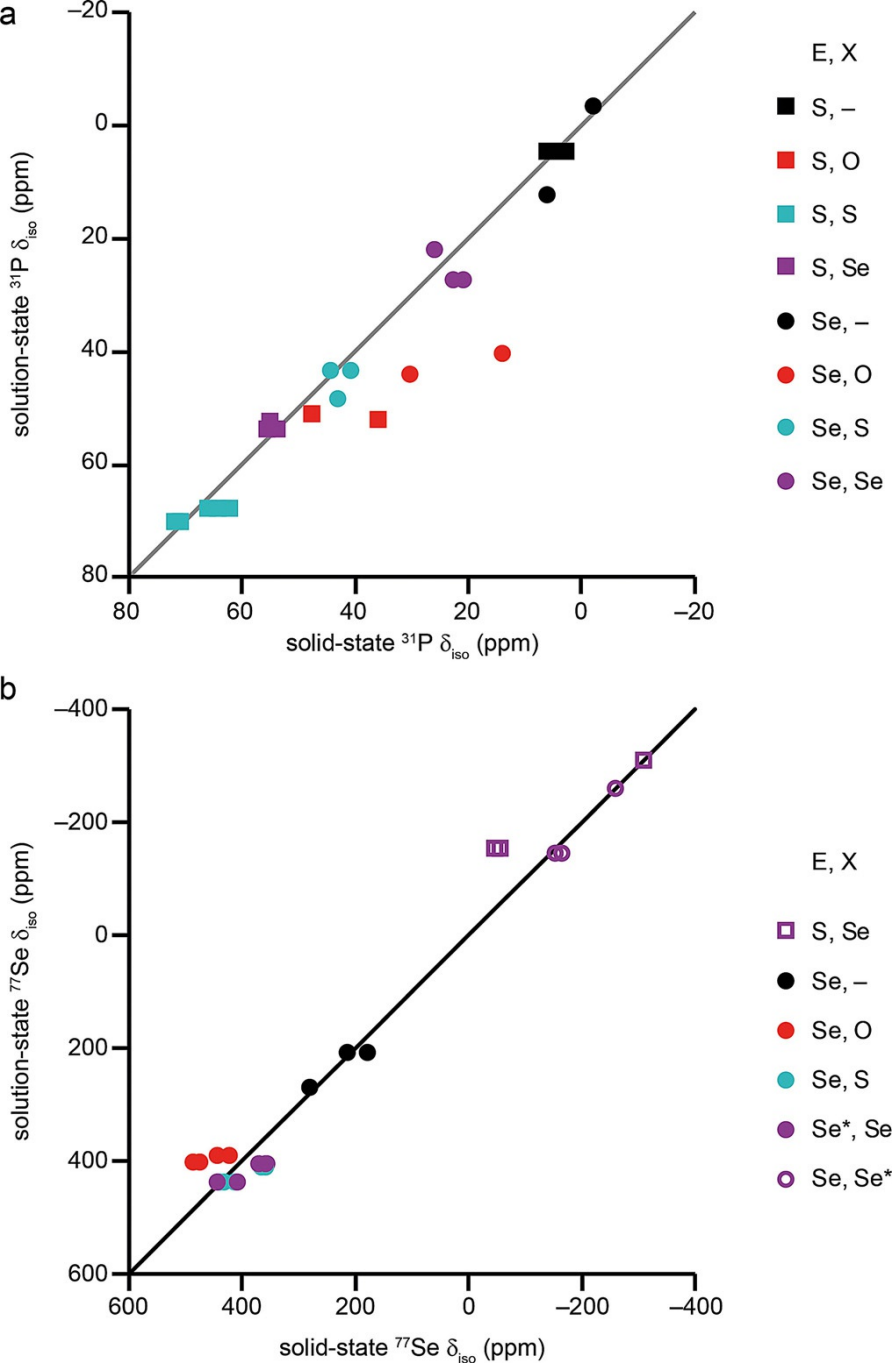
Plots of isotropic chemical shift in solution against those in the solid state for a) ^31^P and b) ^77^Se. In b) the full symbols indicate Se=E and the empty symbols indicate Se=X.

Figure 9 compares the chemical shifts in solution with those determined from the solid‐state NMR spectra for all heterocyclic compounds. For ^31^P (Figure 9 a), the reasonable correlation suggests that the ^31^P chemical shift is primarily determined by the covalent bonding. Generally, both in solution and in the solid state, when E=S (squares), lower *δ*_iso_ is found for unoxidised heterocycles, with an increase on moving from P=O to P=Se and, finally, P=S. For compounds in which E=Se (circles), unoxidised heterocycles again have the lowest *δ*_iso_, with an increase upon =Se oxidation. Larger, but similar, shifts are seen for P=O and P=S compounds. The range of shifts is much greater when E=S than when E=Se. However, Figure 9 a shows that there is a reasonable degree of scatter in the correlation, suggesting that the crystal packing may also affect the values observed in the solid state. Indeed, small variations in isotropic shift are seen between crystallographically distinct ^31^P species in the same material (Table 4) and in different polymorphs of the same compound (Figure 6). Three points lie significantly off the ideal 1:1 correlation shown in Figure 9 a and, notably, all result from P=O compounds (**2**, **6** and **14**). There is a good correlation between solution‐state and solid‐state ^77^Se chemical shifts (Figure 9 b). The smallest chemical shifts are found for P=Se species. For P−Se species, the smallest shifts are seen for unoxidised heterocycles, with oxidation of P increasing *δ*_iso_, although this is similar for most compounds. The points corresponding to **12** lie significantly off the ideal 1:1 correlation, and the shift of the P=Se species is much higher in the solid state than in solution. It is also noticeable that the points from compounds with P=O (**6** and **14**, red circles) all lie slightly above the ideal correlation.

**Figure 10:**
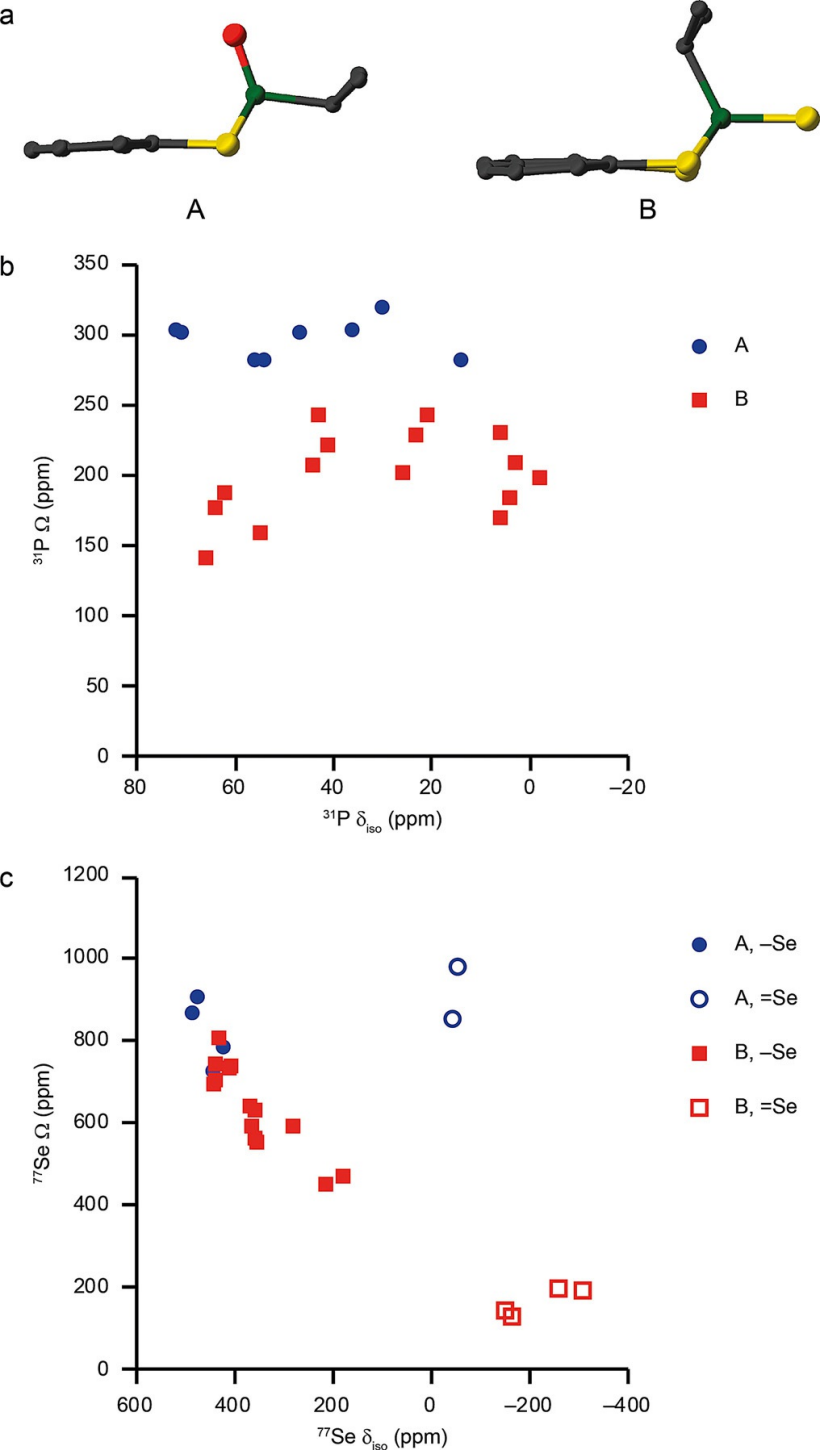
a) Examples of the A and B molecular conformations, as found in **2** and **3 b**. Atoms are coloured with C=grey, P=green, S=yellow and O=red. H atoms are omitted for clarity. b, c) Plots of experimental values of *Ω* against *δ*_iso_ in the solid state for b) ^31^P and c) ^77^Se. In c) the full symbols indicate Se=E and the open symbols indicate Se=X.

As discussed earlier (and as shown in Figure 10 a), two different molecular conformations are adopted in the solid state: A, in which the X‐P‐E_c_‐Nap_c_ dihedral angle (where E_c_ and Nap_c_ are centroids between the E atoms and within the naphthyl group, respectively) is approximately 0°, and B, in which the X‐P‐E_c_‐Nap_c_ dihedral angle is about 180°. Interestingly (and as shown in Section S1 of the Supporting Information), all P=O compounds adopt conformation A. For compounds in which R=*i*Pr (**1**–**9**), only the two P=O compounds (**2** and **6**) adopt this conformation. When R=*t*Bu, all S compounds adopt the A conformation (with the exception of unoxidised heterocycle **9**), while the corresponding Se compounds exhibit the B conformation, with the exception, as stated above, of P=O compound **14**. For compounds that exhibit polymorphism, all polymorphs adopt the same conformation, with variations only in the crystal packing. It would seem, therefore, that some of the differences in chemical shift observed between solid‐ and solution‐state NMR spectra, may result from the fact that two distinct conformations are possible in the solid state (in each case fixed as a result of crystal packing), as opposed to the more dynamic conformational averaging that likely exists in solution. To explore the effect of conformation on the NMR parameters, calculations were performed for (optimised) isolated molecules of **12**, **14** and **16** in both A and B conformations. Values of the isotropic shielding $\sigma_{\mathrm{iso}}^{\mathrm{calcd}}$ and span *Ω* ^calcd^ for ^31^P and ^77^Se are given in Table 5. For ^31^P, small differences in $\sigma_{\mathrm{iso}}^{\mathrm{calcd}}$ (between 1 and 17 ppm) are seen, while for ^77^Se, more significant differences in $\sigma_{\mathrm{iso}}^{\mathrm{calcd}}$ are observed (135–323 ppm for P−Se and 249–345 ppm for P=Se).

**Table 5:** Calculated ^77^Se and ^31^P NMR parameters (isotropic chemical shielding $\sigma_{\mathrm{iso}}^{\mathrm{calcd}}$ and span *Ω* ^calcd^) for isolated molecules of **12**, **14** and **16** in two different conformations.

Species	$\sigma_{\mathrm{iso}}^{\mathrm{calcd}}$ , *Ω* ^calcd^ [ppm]	A	B
**12** (S, *t*Bu, Se)			
^31^P	$\sigma_{\mathrm{iso}}^{\mathrm{calcd}}$	182	193
	*Ω* ^calcd^	491	293
^77^Se (=Se)	$\sigma_{\mathrm{iso}}^{\mathrm{calcd}}$	1628	1877
	*Ω* ^calcd^	1337	225
**14** (Se, *t*Bu, O)			
^31^P	$\sigma_{\mathrm{iso}}^{\mathrm{calcd}}$	214	197
	*Ω* ^calcd^	439	256
^77^Se (−Se)	$\sigma_{\mathrm{iso}}^{\mathrm{calcd}}$	1136	1271
	*Ω* ^calcd^	903	576
**16** (Se, *t*Bu, Se)			
^31^P	$\sigma_{\mathrm{iso}}^{\mathrm{calcd}}$	165	166
	*Ω* ^calcd^	507	295
^77^Se (−Se)	$\sigma_{\mathrm{iso}}^{\mathrm{calcd}}$	909	1232
	*Ω* ^calcd^	1329	721
^77^Se (=Se)	$\sigma_{\mathrm{iso}}^{\mathrm{calcd}}$	1463	1808
	*Ω* ^calcd^	1415	236

Clearly there are more significant variations in *Ω* ^calcd^ between the two conformations for both ^31^P and ^77^Se. For ^31^P, *Ω* ^calcd^ varies from 440–500 in A to 250–300 in B (with a typical difference in one compound of ≈200 ppm). Differences in ^77^Se *Ω* ^calcd^ are more significant, with changes of 300–600 ppm and about 1100 ppm, for P−Se and P=Se species, respectively. Although this information is lost in solution‐state NMR measurements, owing to the rapid tumbling motion of the molecules, Table 4 suggests that solid‐state NMR measurements of *Ω* (particularly for ^77^Se, where possible) can be used to indicate the molecular conformation adopted in the bulk powder. Figure 10 b and c plot experimental *Ω* against *δ*_iso_ for ^31^P and ^77^Se, respectively.

Values of *Ω*, *κ*, and the principal components of the shielding tensors *δ_ii_* are given in Tables S6.1 and S6.2 of the Supporting Information. There is a clear distinction in ^31^P *Ω* (Figure 10 b) for compounds adopting conformation A or conformation B (although a slightly greater spread of *Ω* is seen for B). Although the ^77^Se *Ω* value (Figure 10 c) for P−Se species is generally larger for compounds that adopt the A conformation, there is some overlap of the exhibited ranges of *Ω*. However, there is a very clear distinction for P=Se species for molecules with the A conformation (*Ω*≈800–1000 ppm) and the B conformation (*Ω*≈200 ppm), in excellent agreement with the DFT calculations. As shown in Figure S7.1 of the Supporting Information, there is also good agreement between ^1^ *J*(^31^P−^77^Se) in solution and in the solid state. There is a clear distinction in the magnitude of the *J* coupling seen in the solid state for P−Se species in unoxidised (≈270–300 Hz) and oxidised (≈400 Hz) heterocycles, although the ranges have a little more overlap in solution. The *J* coupling for P=Se species is much greater in both solution and in the solid state, as expected. There are no significant differences in the magnitude of any *J* couplings between molecules adopting the A or B conformations in the solid state.

## Conclusions

The presence of extensive polymorphism in a series of heterocycles was confirmed by using the complementary techniques of single‐crystal and powder XRD, as well as solid‐state NMR spectroscopy. It is clear that although single‐crystal XRD is essential to understand the detailed structure of each polymorph, methods such as powder XRD and solid‐state NMR spectroscopy are required to determine the fraction of each of these in the bulk simple. PXRD can provide a good indication of the polymorphs present in many cases, although for some compounds the PXRD patterns of the different polymorphs are very similar. In other cases, the complex mixture of polymorphs in the bulk material also hinders detailed analysis of the PXRD patterns. In contrast, ^31^P and ^77^Se solid‐state NMR spectra are very sensitive to changes in the local environment, even for polymorphs with structures that are quite similar, as seen for **12**. Furthermore, solid‐state NMR spectroscopy provides a quantitative determination of the fractions of each polymorph in the bulk sample from the relative intensities of the resonances. For the heterocycles studied here, the tendency to exhibit polymorphism increases as the relative content of more polarizable atoms increases, for example, the two compounds that contain three Se atoms, **8** and **16**, exhibit the greatest number of polymorphs.

Two different arrangements of the P=X bond (and consequently the P−C bond) were observed for the oxidised compounds, and it was shown that the NMR parameters (i.e., *δ*_iso_ and *Ω*), particularly for ^77^Se, are very sensitive to the conformation adopted. This was also confirmed by DFT calculations of NMR parameters for isolated molecules of **12**, **14** and **16** adopting the two different conformations. Comparison of the isotropic chemical shifts in solution and in the solid state show differences for some compounds, and the DFT calculations confirmed that these differences do not generally arise as a result of the crystal packing and intermolecular interactions, but probably as a result of the different conformation found in the solid state and the rapid averaging that likely occurs in solution. Intermolecular interactions are observed (in the form of unusual through‐space *J* couplings) for the unoxidised compounds, but these are limited in the oxidised analogues with occupation of the P lone pair.

This work suggests that polymorphism may be more prevalent than previously thought in chalcogen‐containing materials, a fact that will be of vital importance in the development of new molecular materials and will ultimately determine their properties and applications.

## Experimental Section

All syntheses were carried out under an oxygen‐ and moisture‐free nitrogen atmosphere by using standard Schlenk techniques and glassware. Reagents were obtained from commercial sources and used as received. Dry solvents were collected from an MBraun solvent purification system. Elemental analyses were performed by Stephen Boyer at the London Metropolitan University. IR spectra were recorded for solids as KBr discs and oils on KCl plates in the range 4000–300 cm^−1^ with a PerkinElmer System 2000 Fourier transform spectrometer. Electron impact (EI**+**), atmospheric pressure chemical ionisation (APCI+), atmospheric solids analysis probe (ASAP**+**) and nano‐electrospray (NSI) mass spectra were carried out by the EPSRC National Mass Spectrometry Service, Swansea. ^1^H and ^13^C solution‐state NMR spectra were recorded with a Bruker Avance 400 MHz or a Bruker Avance 300 MHz spectrometer with chemical shifts referenced to residual solvent peaks. ^77^Se and ^31^P solution‐state NMR spectra were recorded with a Jeol GSX 270 MHz spectrometer with chemical shifts referenced to external (CH_3_)_2_Se and 85 % H_3_PO_4_, respectively. Assignments of ^13^C and ^1^H NMR spectra were made with the help of ^1^H–^1^H COSY, ^1^H–^13^C HSQC and ^1^H–^13^C HSBC experiments. The naphtho[1,8‐*cd*]1,2‐dithiole and naphtho[1,8‐*cd*]1,2‐diselenole precursors were prepared by literature procedures.^25^ The syntheses of **5** and **13** have been reported elsewhere.^17^

### Naphtho[1,8‐*cd*]1,2‐dithiole isopropylphosphine [NapS_2_P*i*Pr] (1)

A 1 m solution of lithium triethylborohydride (superhydride) in THF (11.2 mL, 11.2 mmol) was added dropwise to a solution of naphtho[1,8‐*cd*]1,2‐dithiole (1.3 g, 6.8 mmol) in THF (100 mL). The mixture was stirred at room temperature for 15 min, after which a solution of dichloroisopropylphosphine (1.5 mL, 10.2 mmol) in THF (10 mL) was added dropwise to the mixture. The resulting mixture was heated to about 66 °C and left overnight. After the solvent was removed in vacuo, the reaction mixture was extracted with hexane (125 mL), washed with distilled water (200 mL) and the organic layer dried with magnesium sulfate and concentrated under reduced pressure. Column chromatography on silica gel (hexane) was performed to afford the purified target compound as a white solid. Crystals suitable for X‐ray diffraction were grown from hexane (1.2 g, 66 %). IR (KBr disc): $\tilde{\nu }$ =2951w, 2916, 2956w, 1548s, 1494s, 1463w, 1360s 1317w, 1232w, 1203vs, 1192s, 1148w, 1082w, 1030s, 888w, 868w, 813vs, 755vs, 639s, 546w, 533w, 508s, 498 cm^−1^ s; ^1^H{^31^P} NMR (300 MHz, CDCl_3_): *δ*=7.8 (dd, ^3^ *J*_HH_=8.3 Hz, ^4^ *J*_HH_=1.2 Hz, 2 H ArH‐4,5) 7.6 (dd, ^3^ *J*_HH_=7.3 Hz, ^4^ *J*_HH_=1.4 Hz, 2 H, ArH‐2,7) 7.4 (t, ^3^ *J*_HH_=7.8 Hz, 2 H, ArH‐3,6) 1.9 (m, 1 H, CH, H9) 1.1 ppm (d, ^3^ *J*_HH_=7.0 Hz, 2×CH_3,6_ H, H10); ^13^C{^1^H} NMR (75.4 MHz, CDCl_3_): *δ*=135.4 (d, ^4^ *J*_CP_=3.1 Hz, C_q_, ArC‐4a) 131.0 (d, ^3^ *J*_CP_=3.2 Hz, 2×CH, ArC‐2,7) 129.6 (s, 2×CH, ArC‐4,5) 127.7 (d, ^3^ *J*_CP_=4.2 Hz, C_q_, ArC‐8a) 125.7 (s, 2×CH, ArC‐3,6) 124.4 (d, ^2^ *J*_CP_=9.0 Hz, 2×C_q_, ArC‐1,8) 28.8 (d, ^1^ *J*_CP_=31 Hz, CH, C‐9) 18.9 ppm (d, ^2^ *J*_CP_=18.5 Hz, 2×CH_3,_ C10); ^31^P{^1^H} NMR (109.3 MHz, CDCl_3_): *δ*=4.72 ppm (s); MS (APCI^+^): *m*/*z* (%) 265.02 (100) [*M*+H]^+^; elemental analysis calcd (%) for C_13_H_13_PS_2_ (264.35): C 59.07, H 4.96; found: C 59.21, H 4.87.

### Naphtho[1,8‐*cd*]1,2‐dithiole isopropylphosphine oxide [NapS_2_P*i*PrO] (2)

Hydrogen peroxide (30 % in water, 0.2 mL, 2.0 mmol) was added to solution of **1** (0.1 g, 0.4 mmol) in dichloromethane (40 mL) and stirring continued for 5 h. Removal of the volatile substances afforded a pale yellow solid. Crystals suitable for X‐ray diffraction were grown by layering a hexane solution of **2** with dichloromethane (0.1 g, 93 %). M.p. 114–118 °C; IR (KBr disc): $\tilde{\nu }$ =2966w, 2362w, 1546w, 1460w, 1365w, 1213vs, 1035s, 877s, 820vs, 758vs, 667s, 571vs, 554vs, 535 cm^−1^ s; ^1^H{^31^P} NMR (300 MHz; CDCl_3_): *δ*=7.8 (dd, ^3^ *J*_HH_=8.3 Hz, ^4^ *J*_HH_=1.2 Hz, 2 H, ArH‐4,5), 7.6 (dd, ^3^ *J*_HH_=7.3 Hz, ^4^ *J*_HH_=1.2 Hz, 2 H, ArH‐2,7), 7.4 (dd, ^3^ *J*_HH_=7.5 Hz, ^3^ *J*_HH_=7.4 Hz, 2 H, ArH‐3,6), 2.3 (m, 1 H, CH, H9), 1.4 ppm (d, ^3^ *J*_HH_=7.2 Hz, 6 H, 2×CH_3_, H10); ^13^C{^1^H} NMR (75.4 MHz; CDCl_3_) *δ*=136.2 (s, C_q_, ArC‐4a), 132.3 (d, ^3^ *J*_CP_=8.3 Hz, 2×CH, ArC‐2,7), 130.1 (s, 2×CH, ArC‐4,5), 127.0 (d, ^3^ *J*_CP_=6.6 Hz, C_q_, ArC‐8a), 126.7 (d, ^2^ *J*_CP_=3.5 Hz, 2×C_q_, ArC‐1,8), 126.4 (s, 2×CH, ArC‐3,6), 34.8 (d, ^1^ *J*_CP_=70 Hz, CH, C9), 15.5 ppm (d, ^2^ *J*_CP_=3.4 Hz, 2×CH_3_, C10); ^31^P{^1^H} NMR (109.3 MHz, CDCl_3_): *δ*=52.0 ppm (s); MS (APCI^+^): *m*/*z* (%) 281.0223 (56) [*M*+H]^+^, 220.9647 (100) [C_10_H_6_PS_2_], 189.9909 (67) [C_10_H_6_PS]^+^; elemental analysis calcd (%) for C_13_H_13_OPS_2_ (280.34): C 55.7, H 4.7; found: C 55.5, H 4.7.

### Naphtho[1,8‐*cd*]1,2‐dithiole isopropylphosphine sulfide [NapS_2_P*i*PrS] (3)

A solution of **1** (0.15 g, 0.56 mmol) and sulfur flowers (0.07 g, 2.34 mmol) in toluene (50 mL) was heated at 110 °C for 48 h. The resulting solution was allowed to cool to room temperature and, after removal of the volatile substances, column chromatography on silica gel hexane/CH_2_Cl_2_ 1:1 was performed to afford a pale pink solid. Crystals suitable for X‐ray diffraction were grown from diethyl ether (0.1 g, 56 %). M.p. 175–177 °C; IR (KBr disc):$\tilde{\nu }$ =2965w, 2922w, 2862w, 1546s, 1494w, 1447w, 1361w, 1326w, 1262s, 1200s, 1092vs, 1031vs, 878w, 817vs, 757vs, 715vs, 614vs, 566s, 483 cm^−1^ w; ^1^H{^31^P} NMR (300 MHz; CDCl_3_): *δ*=7.8 (dd, ^3^ *J*_HH_=8.2 Hz, ^4^ *J*_HH_=1.1 Hz, 2 H, ArH‐4,5), 7.6 (dd, ^3^ *J*_HH_=7.3 Hz, ^4^ *J*_HH_=1.2 Hz, 2 H, ArH‐2,7), 7.4 (t, ^3^ *J*_HH_=8.0 Hz, 2 H, ArH‐3,6), 2.3 (m, 1 H, CH, H9), 1.4 ppm (s, 6 H, 2×CH_3_, H10); ^13^C{^1^H} NMR (75.4 MHz; CDCl_3_): *δ*=136.1 (s, C_q_, ArC‐4a), 131.2 (d, ^3^ *J*_CP_=7.9 Hz, 2×CH, ArC‐2,7), 130.5 (s, 2×CH, ArC‐4,5), 128.1 (d, ^2^ *J*_CP_=4.8 Hz, 2×C_q_, ArC‐1,8), 126.5 (s, 2×CH, ArH‐3,6), 35.6 (d, ^1^ *J*_CP_=47.8 Hz, CH, C9), 15.5 ppm (s, 2×CH_3_, C10); ^31^P{^1^H} NMR (109.3 MHz, CDCl_3_): *δ*=67.2 ppm (s); MS (APCI^+^): *m*/*z* (%) 296.9992 (100) [*M*+H]^+^.

### Naphtho[1,8‐*cd*]1,2‐dithiole isopropylphosphine selenide [NapS_2_P*i*PrSe] (4)

A solution of **1** (0.1 g, 0.6 mmol) and elemental selenium (0.1 g, 0.7 mmol) in toluene (50 mL) was heated at 110 °C and left overnight. The resulting solution was allowed to cool to room temperature and was filtered through a silica plug with hexane (250 mL) and dichloromethane (250 mL). Removal of the volatile substances afforded a pink solid. Crystals suitable for X‐ray diffraction were grown by layering a solution of **4** in dichloromethane with hexane (0.2 g, 93 %). M.p. 191–197 °C; IR (KBr disc): $\tilde{\nu }$ =2964s, 2922w, 1949w, 1546w, 1493w, 1443w, 1360w, 1261vs, 1202s, 1094vs, 1030vs, 877w, 814vs, 819.1vs, 751s, 664s, 564vs, 428 cm^−1^ w; ^1^H{^31^P} NMR (300 MHz; CDCl_3_): *δ*=7.8 (dd, ^3^ *J*_HH_=8.2 Hz, ^4^ *J*_HH_=1.1 Hz, 2 H, ArH‐4,5), 7.6 (dd, ^3^ *J*_HH_=7.3 Hz, ^4^ *J*_HH_=1.2 Hz, 2 H, ArH‐2,7), 7.4 (dd, ^3^ *J*_HH_=8.0 Hz, ^3^ *J*_HH_=7.5 Hz, 2 H, ArH‐3,6), 2.4 (m, 1 H, H9), 1.2 ppm (d, ^3^ *J*_HH_=6.9 Hz, 6 H, 2×CH_3_, H10); ^13^C{^1^H} NMR (75.4 MHz; CDCl_3_): *δ*=136.1 (s, C_q_, ArC‐4a), 130.8 (d, ^3^ *J*_CP_=7.0 Hz, 2×CH, ArC‐2,7), 130.5 (s, 2×CH, ArC‐4,5), 127.8 (d, ^2^ *J*_CP_=5.5 Hz, 2×C_q_, ArC‐1,8), 126.5 (s, 2×CH, ArH‐3,6), 126.3 (d, ^3^ *J*_CP_=7.0 Hz, C_q_, ArC‐8a), 35.2 (d, ^1^ *J*_CP_=37.0 Hz, CH, C9), 16.0 ppm (s, 2×CH_3_, C10); ^31^P{^1^H} NMR (109.3 MHz, CDCl_3_): *δ*=51.6 ppm (s, ^1^ *J* (^31^P–^77^Se)=797 Hz); ^77^Se{^1^H} NMR (51.5 MHz, CDCl_3_): *δ*=−310.6 ppm (d, ^1^ *J* (^31^P–^77^Se)=797 Hz); MS (APCI^+^): *m*/*z* (%) 343.9350 (3) [*M*]^+^, 220.9641 (100) [C_10_H_6_S_2_P]^+^, 189.9904 (82) [C_10_H_6_S_2_]^+^; elemental analysis calcd (%) for C_13_H_13_SePS_2_ (343.3): C 45.5, H 3.8; found: C 45.4, H 3.9.

### Naphtho[1,8‐*cd*]1,2‐diselenole isopropylphosphine oxide [NapSe_2_P*i*PrO] (6)

H_2_O_2_ (30 % solution in water) (0.8 mL, 8.4 mmol) was added dropwise (60 μL per 20 min) to a solution of **5** (0.2 g, 0.7 mmol) in toluene (80 mL) in an ice bath. Stirring was continued until complete consumption of the starting material, monitored by ^31^P NMR spectroscopy. The reaction mixture was washed with water (100 mL), and the organic layer dried with magnesium sulfate and concentrated under reduced pressure. Crystals suitable for X‐ray diffraction were grown by layering a dichloromethane solution of **6** with hexane (0.1 g, 60 %). M.p. 79–82 °C; IR (KBr disc):$\tilde{\nu }$ =3433.6s, 2964s, 1639.5s, 1538.8s, 1460.5s, 1349.6s, 1261.6vs, 1195.6vs, 1096.5vs, 1028.6vs, 874s, 798.3vs, 754.5vs, 653.7s, 498vs, 383.9s, 340.4s, 309.2s, 294.8s, 260.2 cm^−1^ vs; ^1^H{^31^P} NMR (400 MHz; CDCl_3_): *δ*=7.8 (dd, ^3^ *J*_HH_=8.3 Hz, ^4^ *J*_HH_=1.3 Hz, 2 H, ArH‐4,5), 7.7 (dd, ^3^ *J*_HH_=7.3 Hz, ^4^ *J*_HH_=1.3 Hz, 2 H, ArH‐2,7), 7.3 (dd, ^3^ *J*_HH_=8.2 Hz, ^3^ *J*_HH_=7.3 Hz, 2 H, ArH‐3,6), 2.37 (m, CH, H9), 1.4 ppm (d, ^3^ *J*_HH_=7.2 Hz, 3×CH_3_, H10); ^13^C{^1^H} NMR (100.6 MHz; CDCl_3_): *δ*=136.5 (s, C_q_, ArC‐4a), 134.3 (d, ^3^ *J*_CP_=7.7 Hz, 2×CH, ArC‐2,7), 131.0 (s, 2×CH, ArC‐4,5), 128.6 (d, ^2^ *J*_CP_=3.1 Hz, 2×C_q_, ArC‐1,8), 126.4 (s, 2×CH, ArH‐3,6), 38.9 (d, ^1^ *J*_CP_=52.9 Hz, CH, C9), 16.1 ppm (s, 3×CH_3_, C10); ^31^P{^1^H} NMR (109.3 MHz, CDCl_3_): *δ*=40.4 ppm (s, ^1^ *J* (^31^P–^77^Se)=398.4 Hz); ^77^Se{^1^H} NMR (51.5 MHz, CDCl_3_): *δ*=403.8 ppm (d, ^1^ *J* (^31^P–^77^Se)=396.6 Hz); MS (APCI^+^): *m*/*z* (%) 376.9107 (100) [*M*+2 H]^+^, 286.8873 (67) [C_10_H_6_Se_2_+H]^+^; elemental analysis calcd (%) for C_13_H_13_OPSe_2_ (374.1): C 41.7, H 3.5; found: C 41.6, H 3.4.

### Naphtho[1,8‐*cd*]1,2‐diselenole isopropylphosphine sulfide [NapSe_2_P*i*PrS] (7)

A solution of **5** (0.4 g, 1.0 mmol) and elemental sulfur (0.03 g, 1.0 mmol) in toluene (30 mL) was heated at 80 °C for several hours. The resulting solution was allowed to cool to room temperature and then the solvent was removed in vacuo. Column chromatography on silica gel with hexane/dichloromethane (4:1) was performed to afford the purified target compound as a purple solid. Crystals suitable for X‐ray diffraction were grown by layering a dichloromethane solution of **7** in methanol (0.2 g, 61 %). M.p. 147–150 °C; IR (KBr disc): $\tilde{\nu }$ =3424w, 2921w, 1655w, 1539s, 1488w, 1441w, 1357s, 1315w, 1192s, 1032s, 816vs, 753s, 700vs, 590vs, 482s, 384 cm^−1^ w; ^1^H{^31^P} NMR (400 MHz; CDCl_3_): *δ*=7.8 (dd, ^3^ *J*_HH_=8.3 Hz, ^4^ *J*_HH_=1.2 Hz, 2 H, ArH‐4,5), 7.8 (dd, ^3^ *J*_HH_=7.3 Hz, ^4^ *J*_HH_=1.3 Hz, 2 H, ArH‐2,7), 7.4 (dd, ^3^ *J*_HH_=8.1 Hz, ^3^ *J*_HH_=7.2 Hz, 2 H, ArH‐3,6), 2.4 (m, 1 H, CH, H9), 1.3 ppm (m, 6 H, 2×CH_3_, H10); ^13^C{^1^H} NMR (100.6 MHz; CDCl_3_): *δ*=136.3 (s, C_q_, ArC‐4a), 132.9 (d, ^3^ *J*_CP_=6.9 Hz, 2×CH, ArC‐2,7), 131.2 (s, 2×CH, ArC‐4,5), 128.3 (d, ^2^ *J*_CP_=5.5 Hz, 2×C_q_, ArC‐1,8), 128.0 (d, ^3^ *J*_CP_=3.5 Hz, 2×C_q_, ArC‐8a), 126.3 (s, 2×CH. ArC‐3,6) 39.1 (d, ^1^ *J*_CP_=35.1 Hz, P‐CH, C9) 15.8 ppm (s, 2×CH_3_, C10); ^31^P{^1^H} NMR (109.3 MHz, CDCl_3_): *δ*=43.3 ppm (s, ^1^ *J* (^31^P–^77^Se)=385 Hz); ^77^Se{^1^H} NMR (51.5 MHz, CDCl_3_): *δ*=438.7 ppm (d, ^1^ *J* (^31^P–^77^Se)=385 Hz); MS (EI^+^): *m*/*z* (%) 391.9 (15) [*M*+H]^+^, 285.8 (100) [C_10_H_6_Se_2_]^+^, 237.9 (33) [C_10_H_6_SeP]^.+^, 205.9 (33) [C_10_H_6_Se]^.+^, 126.0 (32) [C_10_H_6_]^.+^; elemental analysis calcd (%) for C_13_H_13_SPSe_2_ (390.20): C 40.02, H 3.36; found: C 40.14, H 3.31.

### Naphtho[1,8‐*cd*]1,2‐diselenole isopropylphosphine selenide [NapSe_2_P*i*PrSe] (8)

A solution of **5** (0.5 g, 1.4 mmol) and elemental selenium (0.1 g, 1.7 mmol) in toluene (30 mL) was heated to 80 °C and left overnight. The resulting solution was allowed to cool to room temperature and then the solvent was removed in vacuo. Column chromatography on silica gel with hexane/dichloromethane (4:1) was performed to afford the purified target compound as an orange‐pink solid. Crystals suitable for X‐ray diffraction were grown by layering a dichloromethane solution of **8** in methanol (0.6 g, 77 %). M.p. 150–153 °C; IR (KBr disc): $\tilde{\nu }$ =3450w, 2962w, 2858w, 1539s, 1487w, 1438s, 1355s, 1312w, 1237w, 1191s, 1135w, 1084w, 1027s, 871w, 845w, 812vs, 750vs, 686w, 648vs, 515vs, 475vs, 424w, 374 cm^−1^ w; ^1^H{^31^P} NMR (400 MHz; CDCl_3_): *δ*=7.9 (dd, ^3^ *J*_HH_=8.3 Hz, ^4^ *J*_HH_=1.2 Hz, 2 H, ArH‐4,5), 7.8 (dd, ^3^ *J*_HH_=7.3 Hz, ^4^ *J*_HH_=1.3 Hz, 2 H, ArH‐2,7), 7.4 (dd, ^3^ *J*_HH_=8.1 Hz, ^3^ *J*_HH_=7.3 Hz, 2 H, ArH‐3,6), 2.5 (m, 1 H,CH, H9), 1.2 ppm (d, ^3^ *J*_HH_=6.9 Hz, 6 H, 2×CH_3_, H10); ^13^C{^1^H} NMR (100.6 MHz; CDCl_3_): *δ*=136.3 (s, C_q_, ArC‐4a), 132.5 (d, ^3^ *J*_CP_=6.2 Hz, 2×CH, ArC‐2,7), 131.3 (s, 2×CH, ArC‐4,5), 128.3 (d, ^2^ *J*_CP_=6.1 Hz, 2×C_q_, ArC‐1,8), 127.9 (d, ^3^ *J*_CP_=3.7 Hz, 2×C_q_, ArC‐8a), 126.3 (s, 2×CH, ArC‐3,6), 38.2 (d, ^1^ *J*_CP_=26.3 Hz, P‐CH, C9) 16.3 ppm (s, 2×CH_3_, C10); ^31^P{^1^H} NMR (109.3 MHz, CDCl_3_): *δ*=22.0 ppm (s, ^1^ *J* (^31^P–^77^Se)=391 Hz, ^1^ *J* (^31^P–^77^Se)=773 Hz); ^77^Se{^1^H} NMR (51.5 MHz, CDCl_3_); *δ*=439.2 (d, ^1^ *J* (^31^P–^77^Se)=391 Hz), −260 ppm (d, ^1^ *J* (^31^P–^77^Se)=773 Hz); MS (APCI^+^): *m*/*z* (%): 438.8339 (53) [*M*+H]^+^, 360.9161 (38) [C_13_H_13_PSe_2_]^+^; elemental analysis calcd (%) for C_13_H_13_PSe_3_ (437.1): C 35.7, H 3.0; found: C 35.7, H 2.9.

### Naphtho[1,8‐*cd*]1,2‐dithiole *tert*‐butylphosphine [NapS_2_P*t*Bu] (9)

A 1 m solution of superhydride in THF (14.0 mL, 14.0 mmol) was added dropwise to a solution of naphtho[1,8‐*cd*]1,2‐dithiole (1.3 g, 6.8 mmol) in THF (100 mL). The mixture was stirred at room temperature for 15 min, after which a solution of dichloro‐*tert*‐butylphosphine (1.1 g, 6.83 mmol) in THF (10 mL) was added dropwise to the mixture. The resulting mixture was warmed to about 66 °C and left overnight. After the solvent was removed in vacuo, the reaction mixture was extracted with hexane (125 mL), washed with distilled water (200 mL) and the organic layer dried with magnesium sulfate and concentrated under reduced pressure. Column chromatography on silica gel (hexane) was performed to afford the purified target compound as a yellowish sticky solid. Crystals suitable for X‐ray diffraction were grown from hexane (1.2 g, 72 %). ^1^H{^31^P} NMR (300 MHz, CDCl_3_): *δ*=7.7 (dd, ^3^ *J*_HH_=8.1 Hz, ^4^ *J*_HH_=1.2 Hz, 2H ArH‐4,5) 7.6 (dd, ^3^ *J*_HH_=7.5 Hz, ^4^ *J*_HH_=1.0 Hz, 2 H, ArH‐2,7) 7.3 (t, ^3^ *J*_HH_=7.3 Hz, ^3^ *J*_HH_=7.2 Hz, 2 H, ArH‐3,6) 1.1 ppm (d, ^2^ *J*_CP_=15.7 Hz, 3×CH_3,6_ H, H10); ^13^C{^1^H} NMR (75.4 MHz, CDCl_3_): *δ*=135.2 (d, ^4^ *J*_CP_=2.8 Hz, C_q_, ArC‐4a) 129.8 (d, ^3^ *J*_CP_=2.6 Hz, 2×CH, ArC‐2,7) 129.1 (s, 2×CH, ArC‐4,5) 128.3 (d, ^3^ *J*_CP_=4.4 Hz, C_q_, ArC‐8a) 125.8 (d, ^2^ *J*_CP_=10.3 Hz, 2×C_q_, ArC‐1,8) 125.5 (s, 2×CH, ArC‐3,6) 39.6 (d, ^1^ *J*_CP_=38.8 Hz, CH, C‐9) 27.4 ppm (d, ^2^ *J*_CP_=18.0 Hz, 2×CH_3,_ C10); ^31^P{^1^H} NMR (109.3 MHz, CDCl_3_): *δ*=24.1 ppm (s); MS (EI^+^): *m*/*z* (%) 278.0 (10) [*M* ^⋅^]^+^, 189 (100) [C_10_H_6_S_2_−H]^+^; elemental analysis calcd (%) for C_14_H_15_PS_2_ (278.3): C 60.4, H 5.43; found: C 60.55, H 5.35.

### Naphtho[1,8‐*cd*]1,2‐dithiole *tert*‐butylphosphine oxide [NapS_2_P*t*BuO] (10)

Hydrogen peroxide (30 % in water, 0.1 mL, 9.8 m) was added. to a solution of **9** (0.3 g, 1.0 mmol) in dichloromethane (50 mL) The mixture was stirred overnight to give a yellowish solution. Removal of the volatile substances afforded a pale yellow solid. Crystals suitable for X‐ray diffraction were grown by layering a solution of **10** in dichloromethane with hexane (0.3 g, 93 %). M.p. 230–236 °C (decomp); IR (KBr disc): $\tilde{\nu }$ =2962w, 2926w, 2857w, 2361w, 1546w, 1457w, 1362w, 1262w, 1206vs, 1185vs, 1146s, 883w, 822vs, 761vs, 624vs, 572vs, 510s, 488w, 406 cm^−1^ w; ^1^H{^31^P} NMR (300 MHz; CDCl_3_): *δ*=7.7 (dd, ^3^ *J*_HH_=8.3 Hz, ^4^ *J*_HH_=1.1 Hz, 2 H, ArH‐4,5), 7.6 (dd, ^3^ *J*_HH_=7.3 Hz, ^4^ *J*_HH_=1.2 Hz, 2 H, ArH‐2,7), 7.4 (t, ^3^ *J*_HH_=7.4 Hz, 2 H, ArH‐3,6), 1.4 ppm (s, 9 H, 3×CH_3_, H10); ^13^C{^1^H} NMR (75.4 MHz; CDCl_3_): *δ*=136.1 (s, C_q_, ArC‐4a), 132.5 (d, ^3^ *J*_CP_=7.0 Hz, 2×CH, ArC‐2,7), 130.0 (s, 2×CH, ArC‐4,5), 127.1 (d, ^3^ *J*_CP_=6.3 Hz, C_q_, ArC‐8a), 126.3 (s, 2×CH, ArH‐3,6), 125.6 (d, ^2^ *J*_CP_=4.1 Hz, C_q_, ArC‐1,8), 40.5 (d, ^1^ *J*_CP_=67.6 Hz, C_q_, C9), 24.8 ppm (s, 3×CH_3_, C10); ^31^P{^1^H} NMR (109.3 MHz, CDCl_3_): *δ*=51.7 ppm (s); MS (APCI^+^): *m*/*z* (%) 295.0375 (100) [*M*+H]^+^.

### Naphtho[1,8‐*cd*]1,2‐dithiole *tert*‐butylphosphine sulfide [NapS_2_P*t*BuS] (11)

Compound **11** was prepared by the procedure described previously for **3** by heating **9** (0.27 g, 0.97 mmol) and sulfur flowers (0.04 g, 1.16 mmol) at 90 °C for 72 h. A pinkish orange solid was afforded. Crystals suitable for X‐ray diffraction were grown by layering a solution of **11** in dichloromethane with hexane (0.13 g, 43 %). M.p. 198–203 °C; IR (KBr disc): $\tilde{\nu }$ =2957s, 2922w, 2859s, 1550w, 1495w, 1470w, 1456w, 1365w, 1324w, 1261w, 1203s, 1094br, 1015w, 883w, 813vs, 755vs, 689vs, 601vs, 551vs, 472w; ^1^H{^31^P} NMR (300 MHz; CDCl_3_) δ (ppm)=7.8 (dd, ^3^ *J*_HH_=8.2 Hz, ^4^ *J*_HH_=1.1 Hz, 2 H, ArH‐4,5), 7.6 (dd, ^3^ *J*_HH_=7.3 Hz, ^4^ *J*_HH_=1.2 Hz, 2 H, ArH‐2,7), 7.4 (t, ^3^ *J*_HH_=8.0 Hz, 2 H, ArH‐3,6), 1.4 (s, 9 H, 3×CH_3_, H10); ^13^C{^1^H} NMR (75.4 MHz; CDCl_3_): *δ*=134.8 (s, C_q_, ArC‐4a), 130.0 (d, ^3^ *J*_CP_=7.3 Hz, 2×CH, ArC‐2,7), 129.2 (s, 2×CH, ArC‐4,5), 127.05 (d, ^2^ *J*_CP_=4.9 Hz, 2×C_q_, ArC‐1,8), 125.3 (d, ^3^ *J*_CP_=6.8 Hz, 2×C_q_, ArC‐8a), 125.1 (s, 2×CH, ArC‐3,6), 44.8 (d, ^1^ *J*_CP_=44.1 Hz, C_q_, C9), 24.2 (d, ^2^ *J*_CP_=2.0 Hz, 3×CH_3_, C10); ^31^P{^1^H} NMR (109.3 MHz, CDCl_3_): *δ*=70.2 (s); MS (APCI^+^): *m*/*z* (%) 311.0145 (100) [*M*+H]^+^, 279.0425 (48) [C_14_H_15_PS_2_]^+^; elemental analysis calcd (%) for C_14_H_15_PS_3_ (310.43): C 54.2.1, H 4.9; found: C 53.8, H 5.0.

### Naphtho[1,8‐*cd*]1,2‐dithiole *tert*‐butylphosphine selenide [NapS_2_P*t*BuSe] (12)

Compound **12** was prepared by the procedure described previously for **4**, with **9** (0.3 g, 1.0 mmol) and elemental selenium (0.1 g, 1.1 mmol) yielding a white solid. Crystals suitable for X‐ray diffraction were grown by layering a solution of **12** in dichloromethane with hexane (0.3 g, 97 %). M.p. 203–206 °C; IR (KBr disc): $\tilde{\nu }$ =2964s, 2921w, 1548w, 1494w, 1469w, 1454s, 1364w, 1261vs, 1202s, 1170w, 1094vs, 1016vs, 882w, 812vs, 754s, 614s, 578vs, 548vs, 445 cm^−1^ w; ^1^H{^31^P} NMR (300 MHz; CDCl_3_): *δ*=7.8 (dd, ^3^ *J*_HH_=8.0 Hz, ^4^ *J*_HH_=1.0 Hz, 2 H, ArH‐4,5), 7.6 (dd, ^3^ *J*_HH_=7.3 Hz, ^4^ *J*_HH_=1.0 Hz, 2 H, ArH‐2,7), 7.4 (t, ^3^ *J*_HH_=7.8 Hz, 2 H, ArH‐3,6), 1.4 ppm (s, 9 H, 3×CH_3_, H10); ^13^C{^1^H} NMR (75.4 MHz; CDCl_3_): *δ*=135.9 (s, C_q_, ArC‐4a), 130.6 (d, ^3^ *J*_CP_=6.8 Hz, 2×CH, ArC‐2,7), 130.4 (s, 2×CH, ArC‐4,5), 128.3 (d, ^2^ *J*_CP_=5.5 Hz, 2×C_q_, ArC‐1,8), 126.3 (s, 2×CH, ArH‐3,6), 126.0 (d, ^3^ *J*_CP_=6.8 Hz, C_q_, ArC‐8a), 46.3 (d, ^1^ *J*_CP_=33.0 Hz, C_q_, C9), 25.6 ppm (d, ^2^ *J*_CP_=2.7 Hz, 3×CH_3_, C10); ^31^P{^1^H} NMR (109.3 MHz, CDCl_3_): *δ*=53.8 ppm (s, ^1^ *J* (^31^P–^77^Se)=794 Hz); ^77^Se{^1^H} NMR (51.5 MHz, CDCl_3_): *δ*=−1520.5 ppm (d, ^1^ *J* (^31^P–^77^Se)=794 Hz); MS (APCI^+^): *m*/*z* (%): 358.9588 (100) [*M*+H]^+^, 279.0427 (64) [C_14_H_15_S_2_P]^+^; elemental analysis calcd (%) for C_14_H_15_SePS_2_ (357.3): C 47.1, H 4.2; found: C 47.2, H 4.3.

### Naphtho[1,8‐*cd*]1,2‐diselenole *tert*‐butylphosphine oxide [NapSe_2_P*t*BuO] (14)

H_2_O_2_ (30 % solution in water, 0.14 mL, 1.34 mmol) was added dropwise to a solution of **13** (0.2 g, 0.7 mmol) in dichloromethane (40 mL) and stirring was continued for 1 h. The reaction mixture was washed with water (100 mL), and the organic layer dried with magnesium sulfate and concentrated under reduced pressure. Crystals suitable for X‐ray diffraction were grown from dichloromethane (0.2 g, 88 %). M.p. 199–201 °C; IR (KBr disc): $\tilde{\nu }$ =3422.9s, 2957.2s, 1592.5w, 1541.6s, 1490.2w, 1455.7s, 1362s, 1317.3w, 1196.6vs, 1175vs, 1137.9s, 1008.6w, 819.1vs, 804s, 758.3vs, 689.1w, 616.1s, 505.5vs, 468.3vs, 396.1w, 318.6w, 286.6w, 259.7 cm^−1^ vs; ^1^H{^31^P} NMR (400 MHz; CDCl_3_): *δ*=7.8 (m, 4 H, ArH‐2,7, 4, 5), 7.3 (dd, ^3^ *J*_HH_=8.0 Hz, ^3^ *J*_HH_=7.4 Hz, 2 H, ArH‐3,6), 1.4 ppm (m, 9 H, 3×CH_3_, H10); ^13^C{^1^H} NMR (100.6 MHz; CDCl_3_): *δ*=136.3 (s, C_q_, ArC‐4a), 134.0 (d, ^3^ *J*_CP_=6.9 Hz, 2×CH, ArC‐2,7), 130.8 (s, 2×CH, ArC‐4,5), 128.6 (d, ^2^ *J*_CP_=3.1 Hz, 2×C_q_, ArC‐1,8), 126.3 (s, 2×CH, ArH‐3,6), 44.0 (d, ^1^ *J*_CP_=50.5 Hz, C_q_, C9), 25.0 ppm (s, 3×CH_3_, C10); ^31^P{^1^H} NMR (109.3 MHz, CDCl_3_): *δ*=44.1 ppm (s, ^1^ *J* (^31^P–^77^Se)=406.7 Hz); ^77^Se{^1^H} NMR (51.5 MHz, CDCl_3_): *δ*=392.9 ppm (d, ^1^ *J* (^31^P–^77^Se)=406.7 Hz); MS (APCI^+^): *m*/*z* (%): 390.9268 (28) [*M*+H]^+^, 316.8537 (42) [C_10_H_6_Se_2_P]^+^, 285.8798 (84) [C_10_H_6_Se_2_]^+^, 253.9396 (100) [C_10_H_6_SePO]^+^, 236.9370 (82) [C_10_H_6_SeP]^+^, 206.9710 (31) [C_10_H_6_Se]^+^, 128.0620 (24) [C_10_H_8_]^+^; elemental analysis calcd (%) for C_14_H_15_OPSe_2_ (388.2): C 43.3, H 3.9; found: C 43.2, H 3.8.

### Naphtho[1,8‐*cd*]1,2‐diselenole *tert*‐butylphosphine sulfide [NapSe_2_P*t*BuO] (15)

Compound **15** was prepared by the procedure described previously for **7**, with **13** (0.5 g, 1.3 mmol) and elemental sulfur (0.04 g, 1.4 mmol) yielding a white‐green solid. Crystals suitable for X‐ray diffraction were grown by layering a dichloromethane solution of **15** with hexane (0.4 g, 69 %). M.p. 199–202 °C; IR (KBr disc): $\tilde{\nu }$ =3417w, 2965w, 1638w, 1538w, 1491w, 1455w, 1356s, 1191s, 1013w, 847w, 808s, 750s, 669vs, 581s, 489s, 430 cm^−1^ w; ^1^H{^31^P} NMR (400 MHz; CDCl_3_): *δ*=7.8 (dd, ^3^ *J*_HH_=8.25 Hz, ^4^ *J*_HH_=1.1 Hz, 2 H, ArH‐4,5), 7.8 (dd, ^3^ *J*_HH_=7.3 Hz, ^4^ *J*_HH_=1.2 Hz, 2 H, ArH‐2,7), 7.4 (dd, ^3^ *J*_HH_=8.0 Hz, ^3^ *J*_HH_=7.4 Hz, 2 H, ArH‐3,6), 1.3 ppm (s, 9 H, 3×CH_3_, H10); ^13^C{^1^H} NMR (100.6 MHz; DMSO): *δ*=135.8 (s, C_q_, ArC‐4a), 131.3 (d, ^3^ *J*_CP_=6.6 Hz, 2×CH, ArC‐2,7), 130.7 (s, 2×CH, ArC‐4,5), 128.9 (d, ^2^ *J*_CP_=5.9 Hz, 2×C_q_, ArC‐1,8,), 126.8 (d, ^3^ *J*_CP_=3.1 Hz, 2×C_q_, ArC‐8a), 125.8 (s,2×CH, ArC‐3,6), 47.6 (d, ^1^ *J*_CP_=30.3 Hz, C9), 24.2 ppm (s, 3×CH_3_, C10); ^31^P{^1^H} NMR (109.3 MHz, CDCl_3_): *δ*=48.6 ppm (s, ^1^ *J* (^31^P–^77^Se)=398 Hz); ^77^Se{^1^H} NMR (51.5 MHz, CDCl_3_): *δ*=413.2 ppm (d, ^1^ *J* (^31^P–^77^Se)=398 Hz); MS (EI^+^): *m*/*z* (%): 405.8 (44) [*M*]^.+^, 285.8 (100) [C_10_H_6_Se_2_]^.+^, 205.9 (28) [C_10_H_6_Se]^.+^, 126.0 (40) [C_10_H_6_^⋅^]^+^; elemental analysis calcd (%) for C_14_H_15_SPSe_2_ (404.23): C 41.6, H 3.7; found: C 41.7, H 3.6.

### Naphtho[1,8‐*cd*]1,2‐diselenole *tert*‐butylphosphine selenide [NapSe_2_P*t*BuSe] (16)

Compound **16** was prepared by the procedure described previously for **8**, with **13** (0.2 g, 1.1 mmol) and elemental selenium (0.1 g, 1.4 mmol) yielding a light purple solid. Crystals suitable for X‐ray diffraction were grown by layering a dichloromethane solution of **16** with methanol (0.4 g, 78 %). M.p. 182–185 °C; IR (KBr disc): $\tilde{\nu }$ =3423.7s, 2967s, 2283.9w, 1537.6s, 1490.6w, 1453.4s, 1355.6s, 1327.7s, 1190.3s, 1164.6s, 1012.3s, 846.1w, 807.8vs, 749.1vs, 593.7s, 556.4w, 534.8vs, 520.7vs, 482.2s, 417.4s, 375.4 cm^−1^ s; ^1^H{^31^P} NMR (400 MHz; CDCl_3_): *δ*=7.9 (dd, ^3^ *J*_HH_=8.3 Hz, ^4^ *J*_HH_=1.2 Hz, 2 H, ArH‐4,5), 7.8 (dd, ^3^ *J*_HH_=7.3 Hz, ^4^ *J*_HH_=1.3 Hz, 2 H, ArH‐2,7), 7.4 (dd, ^3^ *J*_HH_=8.1 Hz, ^3^ *J*_HH_=7.3 Hz, 2 H, ArH‐3,6), 1.3 ppm (s, 9 H, 3×CH_3_, H10); ^13^C{^1^H} NMR (100.6 MHz; CDCl_3_): *δ*=136.1 (s, C_q_, ArC‐4a), 131.1 (s, 2×CH, ArC‐4,5), 130.8 (d, ^3^ *J*_CP_=6.01 Hz, 2×CH, ArC‐2,7), 130.3 (d, ^2^ *J*_CP_=6.5 Hz, 2×C_q_, ArC‐1,8), 127.4 (d, ^3^ *J*_CP_=3.5 Hz, 2×C_q_, ArC‐8a), 126.1 (s, 2×CH. ArC‐3,6) 48.2 (d, ^1^ *J*_CP_=20.1 Hz, P‐C_q_, C9) 25.3 ppm (s, 3×CH_3_, C10); ^31^P{^1^H} NMR (109.3 MHz, CDCl_3_): *δ*=27.3 ppm (s, ^1^ *J* (^31^P–^77^Se)=407 Hz, ^1^ *J* (^31^P–^77^Se)=752 Hz); ^77^Se{^1^H} NMR (51.5 MHz, CDCl_3_): *δ*=406.1 (d, ^1^ *J* (^31^P–^77^Se)=407 Hz), −143.7 ppm (d, ^1^ *J* (^31^P–^77^Se)=752 Hz); MS (NSI^+^): *m*/*z* (%): 919.7195 (10) [2 M+NH_4_−H] 452.8489 (100) [*M*+H]^+^; elemental analysis calcd (%) for C_14_H_15_PSe_3_ (453.8): C 37.3, H 3.3; found: C 37.4, H 3.3.

### Crystal structure analyses

X‐ray diffraction data for **1 a**, **8 a**, **9** and **13** were collected at −148(1) °C by using a Rigaku MM007 High‐Brilliance RA generator (Mo_Kα_ radiation, confocal optics) and Saturn CCD system. At least a full hemisphere of data was collected by using *ω* scans. Data for **1 b**, **2**, **3 a**, **4 a**, **6**, **7**, **10**, **12 a**, **14**, **15** and **16 a** were collected at −100(1) °C, and those for **1 c**, **3 b**, **3 c**, **4 b** and **16 b** at −180(1) °C by using a Rigaku FR‐X Ultrahigh‐Brilliance Microfocus RA generator (Mo_Kα_ radiation, confocal optics) with XtaLAB P200 diffractometer. At least a full hemisphere of data was collected by using *ω* scans. Data for **5** were collected at −180(1) °C by using a Rigaku MM007 High Brilliance RA generator (Mo_Kα_ radiation, confocal optics) and Mercury CCD system. At least a full hemisphere of data was collected by using both *ω* and *φ* scans. Data for **8 b** were collected at −100(1) °C by using a Rigaku SCXmini CCD diffractometer (Mo_Kα_ radiation, SHINE monochromator). At least a full hemisphere of data was collected by using *ω* scans. Data for **11** and **12 b** were collected at −148(1) °C by using the St Andrews Automated Robotic Diffractometer (STANDARD),^26^ a Rigaku sealed‐tube generator (Mo_Kα_ radiation, SHINE monochromator) and Saturn 724 CCD system, coupled with a Microglide goniometer head and an ACTOR‐SM robotic sample changer. Data for all compounds were collected and processed (including correction for Lorentzian effects, polarisation and absorption) with CrystalClear (Rigaku).^27^ Structures were solved by direct (SHELXS‐97, −2013,^28^ SIR2004^29^ or SIR2011^30^), charge‐flipping (Superflip^31^) or Patterson (PATTY^32^) methods and expanded by using Fourier techniques. Non‐hydrogen atoms were refined anisotropically. Hydrogen atoms were refined using the riding model. All calculations were performed using the CrystalStructure^33^ crystallographic software package except for refinement, which was performed using SHELXL2013.^34^

https://www.ccdc.cam.ac.uk/services/structures?id=doi:10.1002/chem.201800978 1816237 (**1 a**), 1816238 (**1 b**), 1816239 (**1 c**), 1816236 (**2**), 1816241 (**3 a**), 1816235 (**3 b**), 1816246 (**3 c**), 1816243 (**4 a**), 1816245 (**4 b**), 1057058 (**5**), 1816242 (**6**), 1816248 (**7**), 1816249 (**8 a**), 1816244 (**8 b**), 1816257 (**9**), 1816247 (**10**), 1816252 (**11**), 1816251 (**12 a**), 1816254 (**12 b**), 1057057 (**13**), 1816253 (**14**), 1816255 (**15**), 1816256 (**16 a**), and 1816258 (**16 b**) contain the supplementary crystallographic data for this paper. These data can be obtained free of charge from http://www.ccdc.cam.ac.uk/.

### Solid‐state NMR spectroscopy

Solid‐state NMR measurements were performed with Bruker Avance III spectrometers operating at magnetic field strengths of 9.4 and 14.1 T. Experiments were carried out with conventional 4, 1.9 or 1.3 mm MAS probes at MAS rates between 5 and 55 kHz. For ^31^P, MAS NMR spectra were acquired at 298 K, 14.1 T and 7.5 kHz MAS with ^1^H decoupling. For variable‐temperature experiments the sample temperature was controlled with a Bruker BCU‐II chiller and Bruker BVT/BVTB‐3000 temperature controller and heater booster. The sample temperature was calibrated by using the temperature‐dependent shift of an external sample of RbCl.^35^ Chemical shifts were referenced to 85 % H_3_PO_4_ (aq.) at 0 ppm, by using BPO_4_ at −29.6 ppm as a secondary reference. For ^77^Se, CP MAS experiments (with ramped contact pulse durations of 5–8 ms and TPPM ^1^H decoupling) were carried out at 298 K at 9.4 and 14.1 T. Chemical shifts were referenced to (CH_3_)_2_Se at 0 ppm, by using the isotropic resonance of solid H_2_SeO_3_ at 1288.1 ppm as a secondary reference. The position of the isotropic resonances within the spinning sideband manifolds were unambiguously determined by acquiring a second spectrum at a different MAS rate. In some cases, spectra were also acquired with additional ^31^P continuous wave decoupling. Experimental NMR parameters were determined by line‐shape analysis with Bruker Topspin software, SOLA.

### Computational details

Calculations of *J* coupling were carried out with the CASTEP DFT code (version 17.52),^36^, ^37^ by employing the gauge‐including projector‐augmented wave (GIPAW) algorithm^38^ for the reconstruction of the all‐electron wave function in the presence of a magnetic field. The generalised gradient approximation (GGA) PBE functional^39^ was employed and core‐valence interactions were described by ultrasoft pseudopotentials.^40^ All calculations were performed with the D2 dispersion‐correction scheme of Grimme,^41^ a plane‐wave energy cut‐off of 50 Ry (680 eV) and a *k*‐point spacing^42^ of 0.04×2π Å^−1^. For all calculations, the initial atomic positions and unit‐cell parameters were taken from the single‐crystal X‐ray diffraction structures determined in this work. Prior to the calculation of NMR parameters, geometry optimisations were performed for each structure. All atomic positions and lattice parameters were allowed to vary. All *J* coupling constant were tested for convergence with supercell size by constructing supercells based on the optimised unit cells; for **1 a**, **1 b**, **1 c**, **5** and **13** a 2×1×1 supercell was adopted, while for **9** a 1×2×1 supercell was used. Calculations performed on isolated molecules in alternative conformations were carried out by using CASTEP 7 (PBE, 50 Ry, 0.04×2π Å^−1^, ultrasoft pseudopotentials and D2 dispersion correction). Models were produced by extracting a single molecule from the unit cell and placing it in a 20 Å periodic box before geometry optimisation of all atomic positions. For the alternative X‐P‐E_c_‐Nap_c_ dihedral angle (see Results and Discussion) a 180° rotation about this dihedral angle was performed manually and the geometry reoptimised. Calculations were performed on two computing clusters at the University of St Andrews; single‐molecule calculations used a cluster of 300×12‐core Intel Westmere nodes connected via QDR Infiniband, and more resource‐intensive calculations were performed on a cluster of 54× 32‐core Intel Broadwell nodes with FDR Infiniband interconnect and 300 TB distributed file system (GPFS).

## Conflict of interest

The authors declare no conflict of interest.

## Supporting information

As a service to our authors and readers, this journal provides supporting information supplied by the authors. Such materials are peer reviewed and may be re‐organized for online delivery, but are not copy‐edited or typeset. Technical support issues arising from supporting information (other than missing files) should be addressed to the authors.

Supplementary
