# Supplementary material for: Polymorphism, Weak Interactions and Phase Transitions in Chalcogen–Phosphorus Heterocycles
Source: Chemistry. 2018 Jun 28;24(43):11067–81. doi: 10.1002/chem.201800978 (PMC6099295; doi:10.1002/chem.201800978)
Supplement: Supplementary file 1 — Supplementary [file CHEM-24-11067-s001.pdf]

CHEMISTRY
A European Journal

Supporting Information

Polymorphism, Weak Interactions and Phase Transitions in Chalcogen–Phosphorus Heterocycles

Paula Sanz Camacho, Martin W. Stanford, David McKay, Daniel M. Dawson,
Kasun S. Athukorala Arachchige, David B. Cordes, Alexandra M. Z. Slawin, J. Derek Woollins,*
and Sharon E. Ashbrook*[a]

chem_201800978_sm_miscellaneous_information.pdf

Supporting Information

S1. Summary of conformations and isomorphous relationships

S2. Additional information for unoxidised heterocycles

S3. Additional information for P=O oxidised heterocycles

S4. Additional information for P=S oxidised heterocycles

S5. Additional information for P=Se oxidised heterocycles

S6. Experimental shielding tensors

S7. Comparison of calculated and experimental J couplings

S1. Summary of conformations and isomorphous relationships

Table S1 summarises the conformations and isomorphous relationships of **1-16**. As shown in Figure S1.1 (for **2** and **3b**), molecules adopt two different conformations in the solid state: A, where the P=X bond is aligned almost perpendicular with the plane of the naphthalene ring (and therefore the P-C bond lies parallel to this plane); B where the P=X bond lies parallel to the plane of the naphthalene ring (and the P-C bond is found almost perpendicular to this).

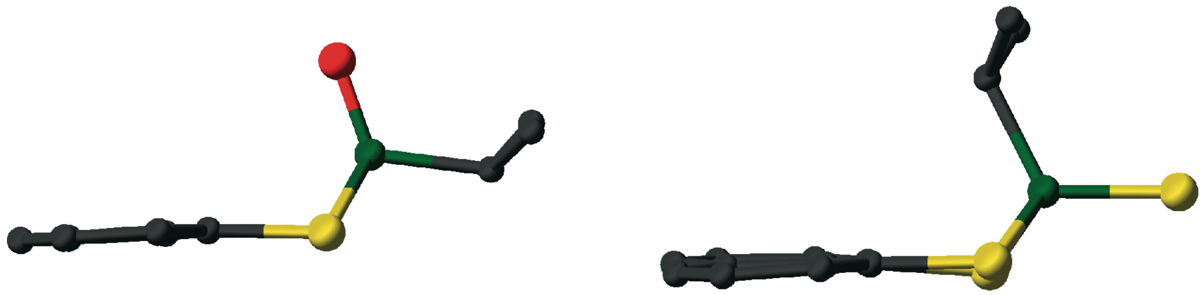

A B

Figure S1.1. Examples of the A and B molecular conformations, as found in **2** and **3b**. Atoms are coloured with C = grey, P = green, S = yellow and O = red. H atoms are omitted for clarity.

Table S1. Conformations and isomorphous relationships for **1-16**.

Compound	E, R, X	No. of crystallographically independent molecules	Conformation	Isomorphous relationships	CCDC
**1a**	S, ^{i}Pr, -	2	B		1816237
**1b**	S, ^{i}Pr, -	4	B		1816238
**1c**	S, ^{i}Pr, -	2	B		1816239
**2**	S, ^{i}Pr, O	1	A	Iso. with **6**	1816236
**3a**	S, ^{i}Pr, S	2	B		1816241
**3b**	S, ^{i}Pr, S	1	B	Iso. with **8a**	1816235
**3c**	S, ^{i}Pr, S	2	B	Iso. with **7, 8b**	1816246
**4a**	S, ^{i}Pr, Se	1	B		1816243
**4b**	S, ^{i}Pr, Se	2	B		1816245
**5**	Se, ^{i}Pr, -	1	B		1057058
**6**	Se, ^{i}Pr, O	1	A	Iso. with **2**	1816242
**7**	Se, ^{i}Pr, S	2	B	Iso. with **3c, 8b**	1816248
**8a**	Se, ^{i}Pr, Se	1	B	Iso. with **3b**	1816249
**8b**	Se, ^{i}Pr, Se	2	B	Iso. with **3c, 7**	1816244
**9**	S, ^{t}Bu, -	2	B		1816257
**10**	S, ^{t}Bu, O	1	A		1816247
**11**	S, ^{t}Bu, S	2	A	Iso. with **12b**	1816252
**12a**	S, ^{t}Bu, Se	2	A		1816251
**12b**	S, ^{t}Bu, Se	2	A	Iso. with **11**	1816254
**13**	Se, ^{t}Bu, -	1	B		1057057
**14**	Se, ^{t}Bu, O	1	A		1816253
**15**	Se, ^{t}Bu, S	1	B	Iso. with **16a**	1816255
**16a**	Se, ^{t}Bu, Se	1	B	Iso. with **15**	1816256
**16b**	Se, ^{t}Bu, Se	2	B		1816258

S2. Additional information for unoxidised heterocycles

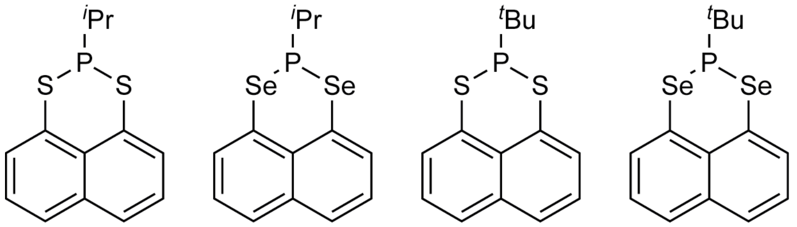

1 **5** **9** **13**

Scheme 2.1. Unoxidised heterocycles.

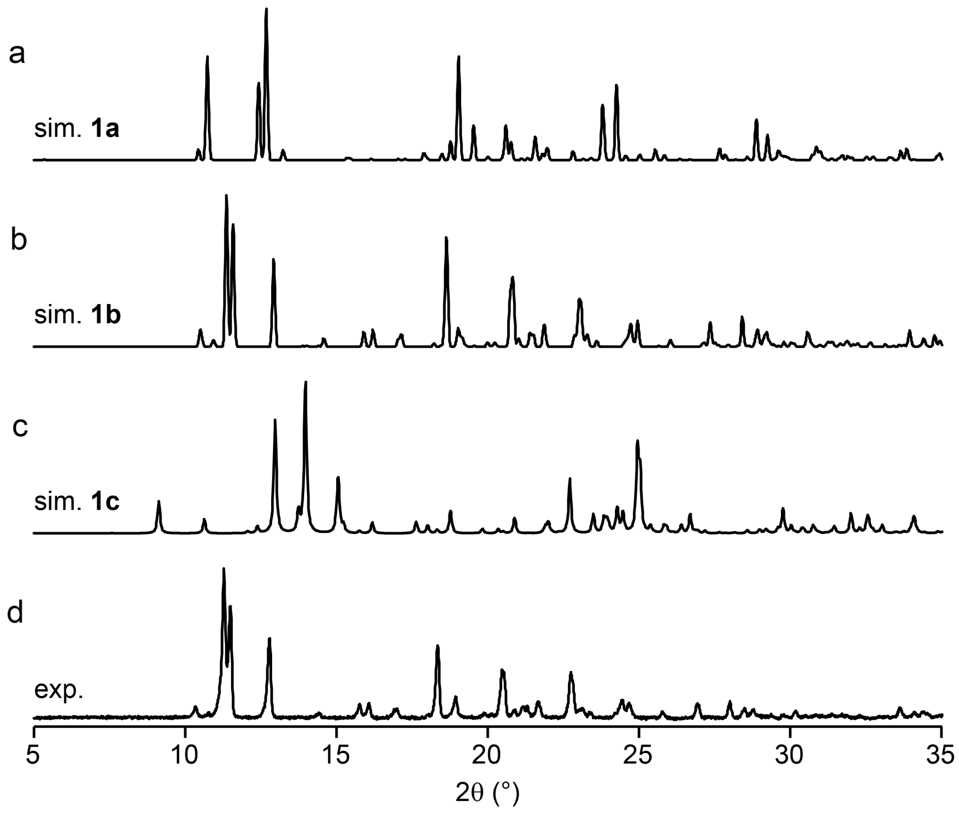

Figure S2.1. Simulated PXRD patterns from crystal structures of (a) **1a**, (b) **1b** and (c) **1c**. (d) Experimental PXRD pattern from the bulk sample of **1**.

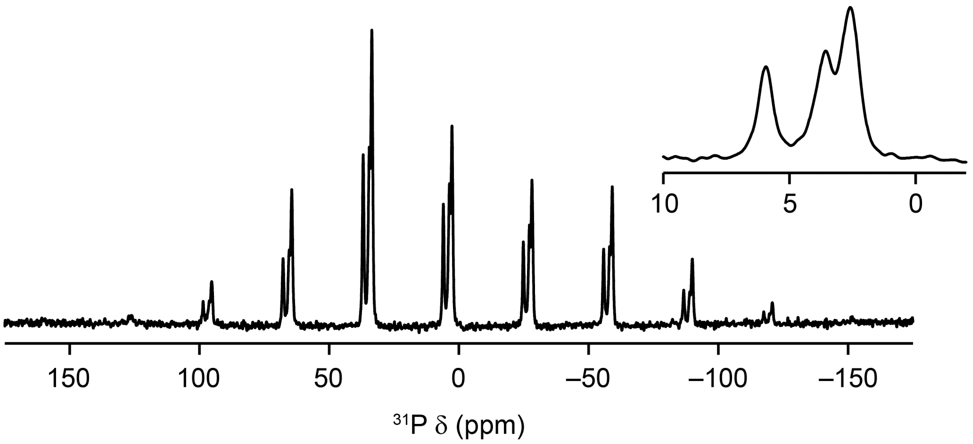

Figure S2.2. ^{31}P (14.1 T, 7.5 kHz) MAS NMR spectrum of **1**, with an expansion of the isotropic region.

S3. Additional information for P=O oxidised heterocycles

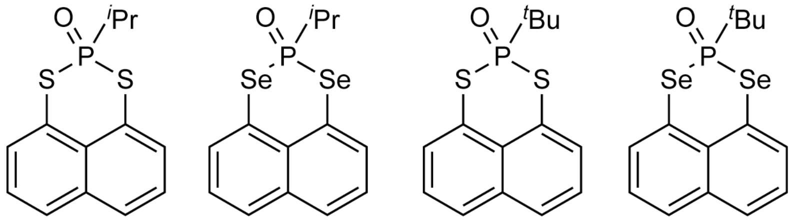

2 **6** **10** **14**

Scheme 3.1. P=O oxidised heterocycles.

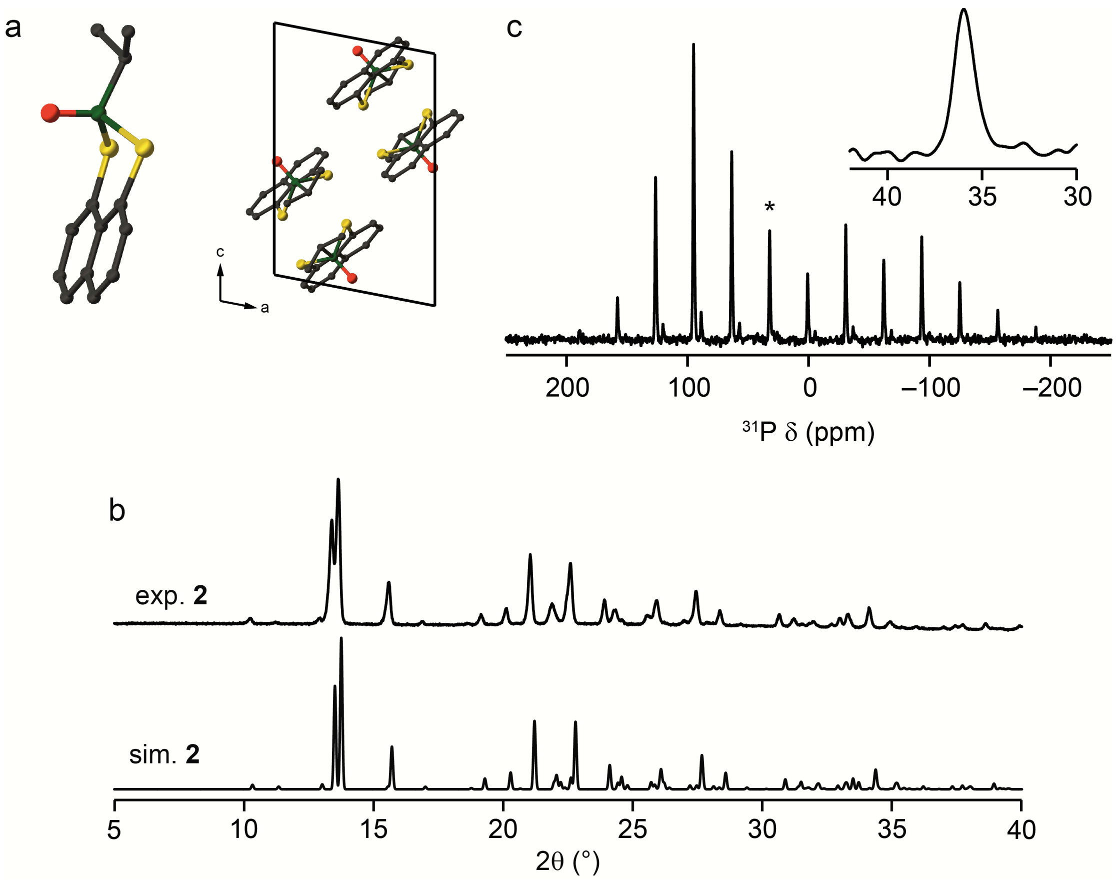

Figure S3.1. (a) Asymmetric unit and packing motif, (b) comparison of the experimental and simulated PXRD patterns and (c) ^{31}P (14.1 T, 7.5 kHz) MAS NMR spectrum for **2**. In (a), atoms are coloured with C = grey, P = green, S = yellow and O = red. H atoms are omitted for clarity. In (c), the isotropic resonance (marked with *) is expanded in the inset.

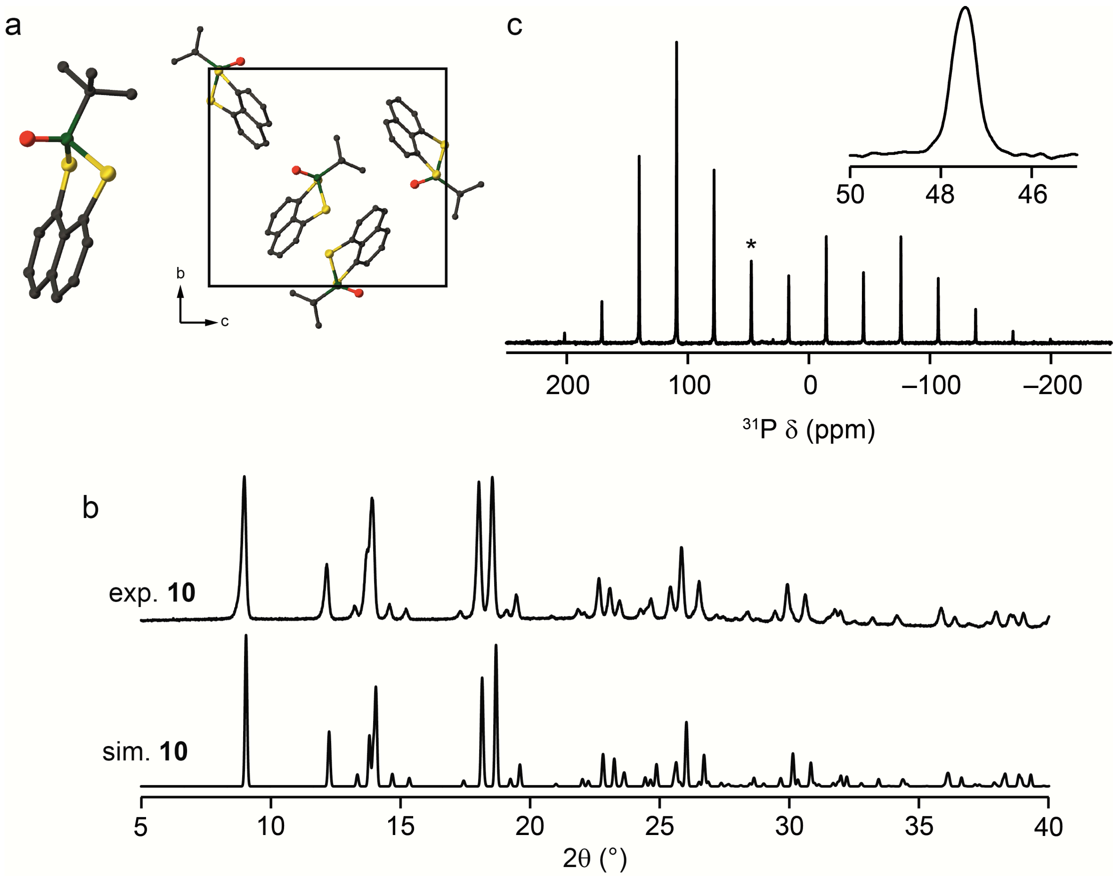

Figure S3.2. (a) Asymmetric unit and packing motif, (b) comparison of the experimental and simulated PXRD patterns and (c) ^{31}P (14.1 T, 7.5 kHz) MAS NMR spectrum for **10**. In (a), atoms are coloured with C = grey, P = green, S = yellow and O = red. H atoms are omitted for clarity. In (c), the isotropic resonance (marked with *) is expanded in the inset.

S4. Additional information for P=S oxidised heterocycles

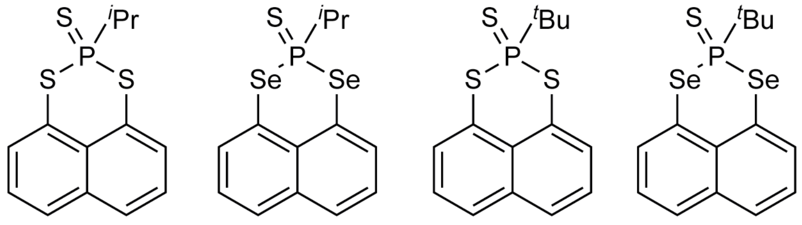

3 **7** **11** **15**

Scheme 4.1. P=S oxidised heterocycles.

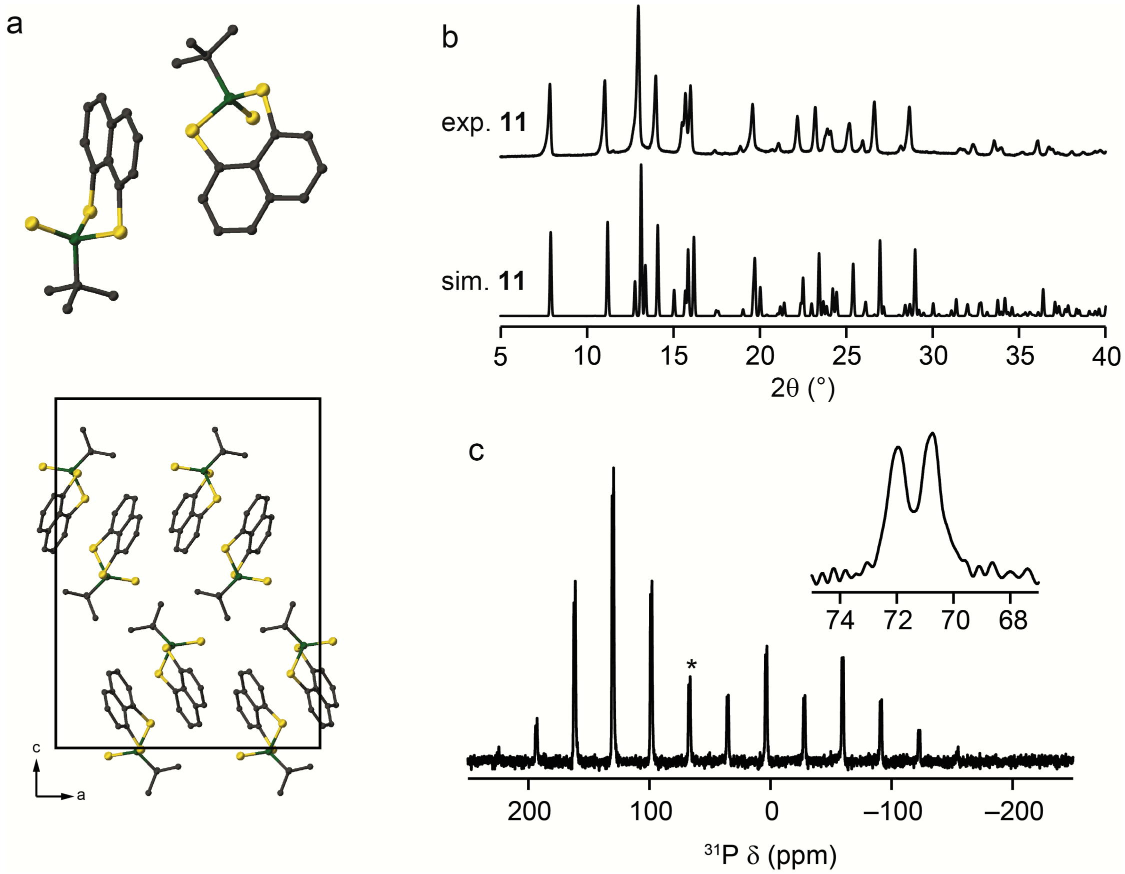

Figure S4.1. (a) Asymmetric unit (black square) and packing motif, (b) comparison of the experimental and simulated PXRD patterns and (c) ^{31}P (14.1 T, 7.5 kHz) MAS NMR spectrum for **11**. In (a), atoms are coloured with C = grey, P = green and S = yellow. H atoms are omitted for clarity. In (c), the isotropic resonances (marked with *) are expanded in the inset.

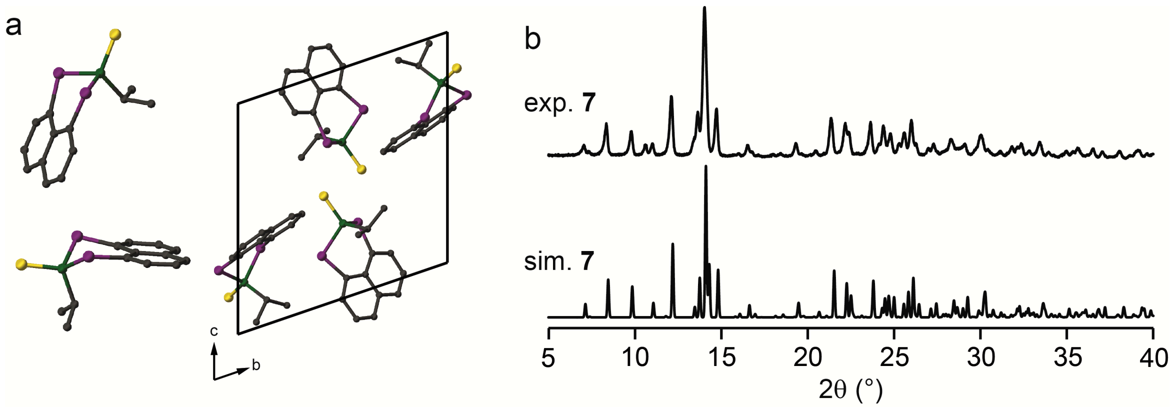

Figure S4.2. (a) Asymmetric unit and packing motif and (b) comparison of the experimental and simulated PXRD patterns of **7**. Atoms are coloured with C = grey, P = green, S = yellow and Se = purple. H atoms are omitted for clarity.

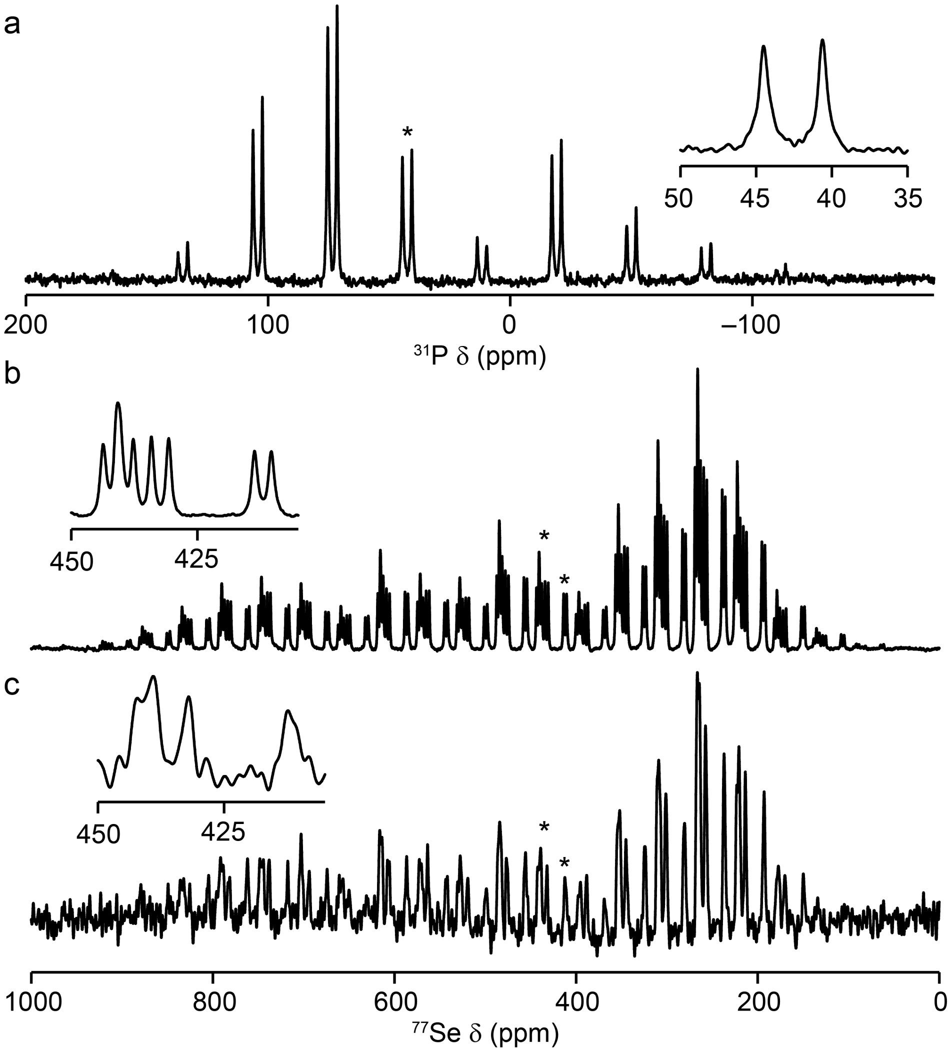

Figure S4.3. (a) ^{31}P (14.1 T, 7.5 kHz) MAS NMR spectrum, (b, c) ^{77}Se (14.1 T, 5 kHz) CP MAS NMR spectra without (b) and with (c) ^{31}P decoupling for **7**. The isotropic resonances (marked with *) are expanded as insets.

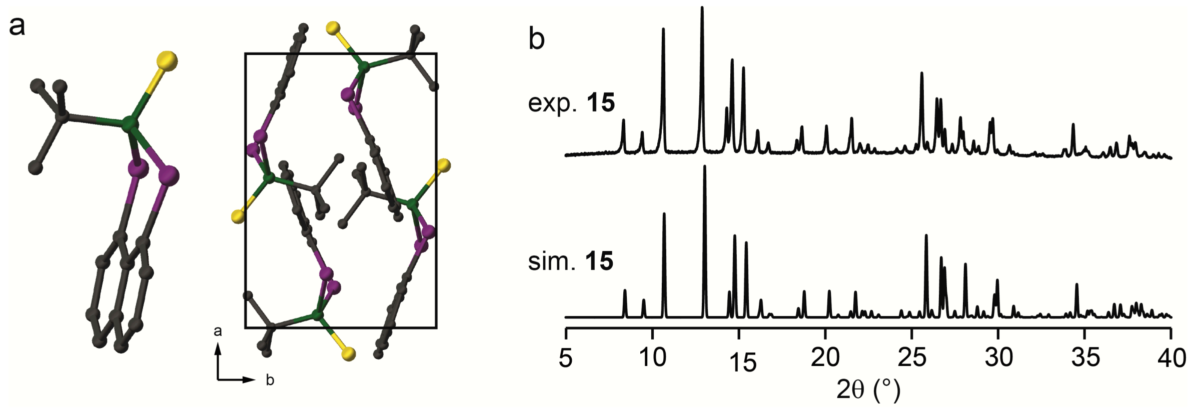

Figure S4.4. (a) Asymmetric unit (black square) and packing motif and (b) comparison of the experimental and simulated PXRD patterns of **15**. Atoms are coloured with C = grey, P = green, S = yellow and Se = purple. H atoms are omitted for clarity.

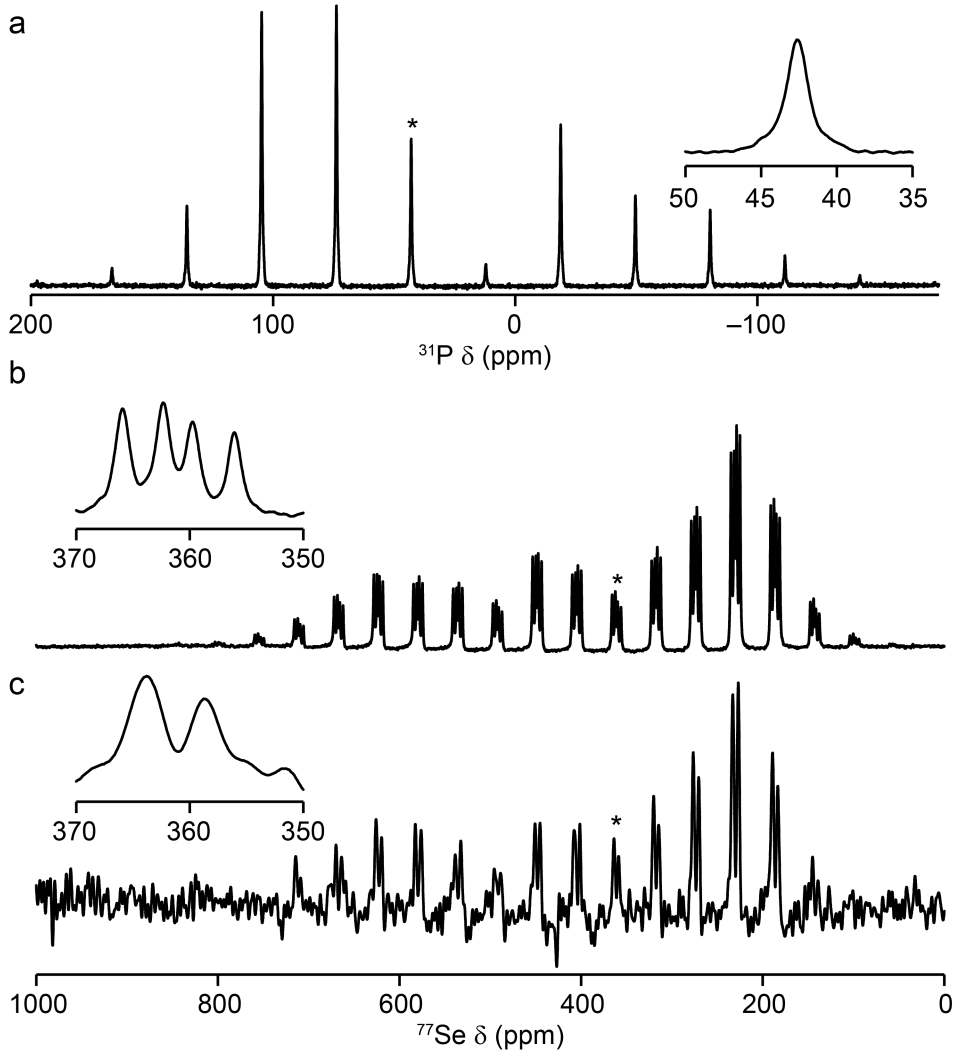

Figure S4.5. (a) ^{31}P (14.1 T, 7.5 kHz) MAS NMR spectrum, (b, c) ^{77}Se (14.1 T, 5 kHz) CP MAS NMR spectra without (b) and with (c) ^{31}P decoupling for **15**. The isotropic resonances (marked with *) are expanded as insets.

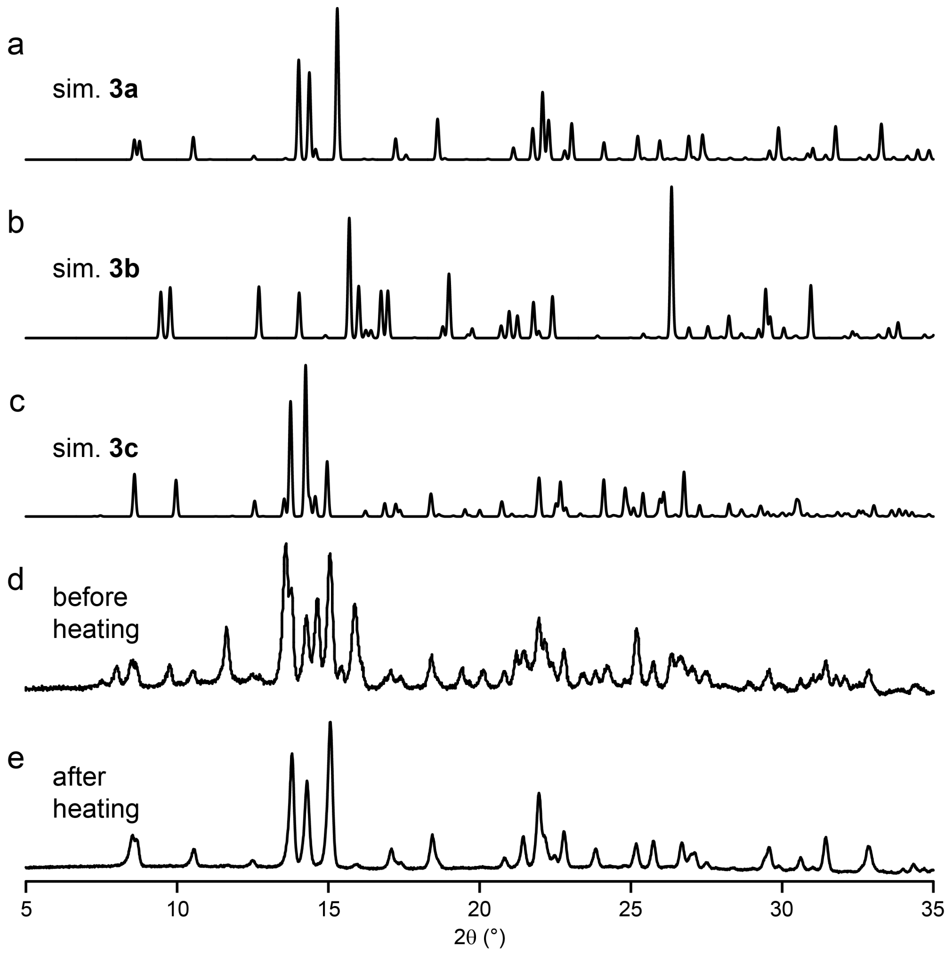

Figure S4.6. Comparison of simulated PXRD patterns for (a) **3a**, (b) **3b** and (c) **3c**, with experimental PXRD of the bulk sample of **3** (d) before and (e) after the VT NMR experiment.

S5. Additional information for P=Se oxidised heterocycles

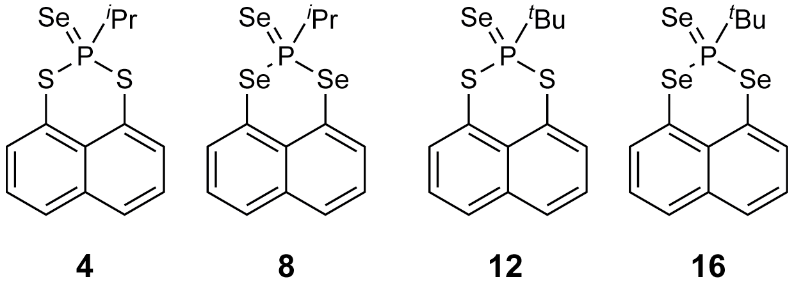

Scheme 5.1. P=Se oxidised heterocycles.

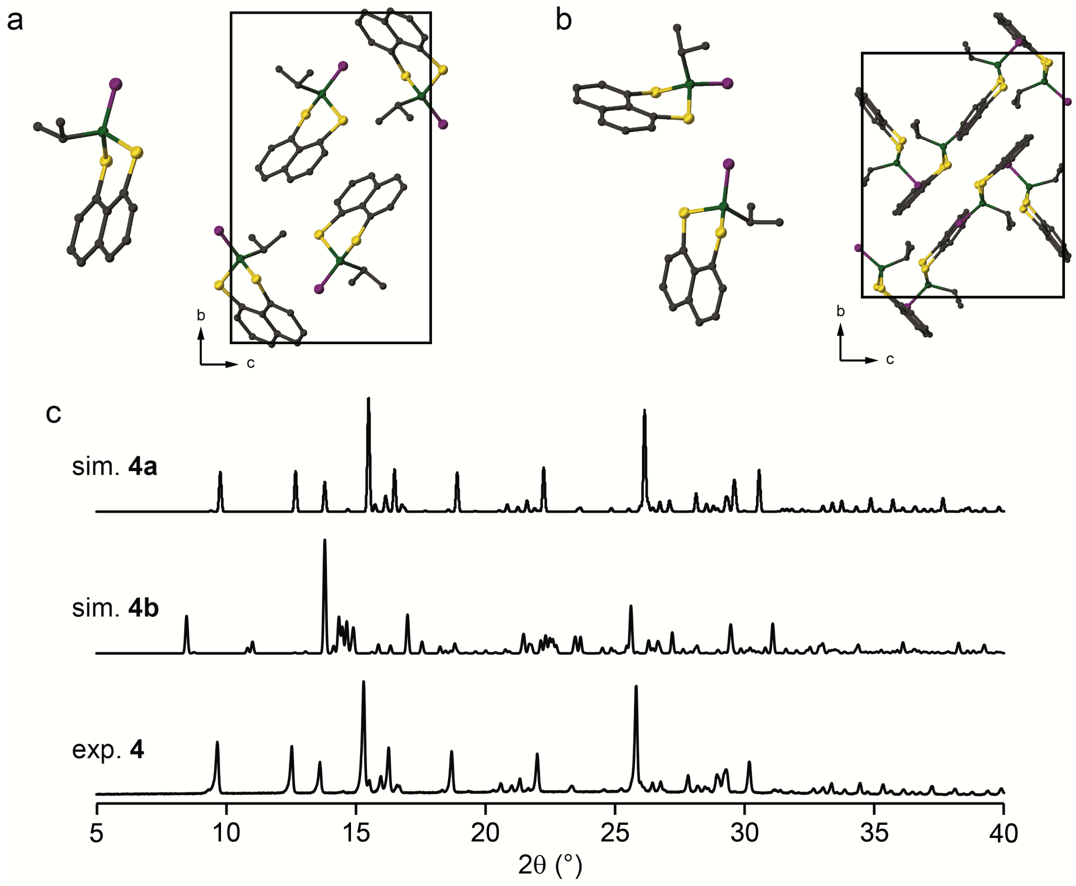

Figure S5.1. (a, b) Asymmetric units and packing motifs for (a) **4a** and (b) **4b**. (c) Comparison of the experimental and simulated PXRD patterns for **4**. Atoms are coloured with C = grey, P = green, S = yellow and Se = purple. H atoms are omitted for clarity.

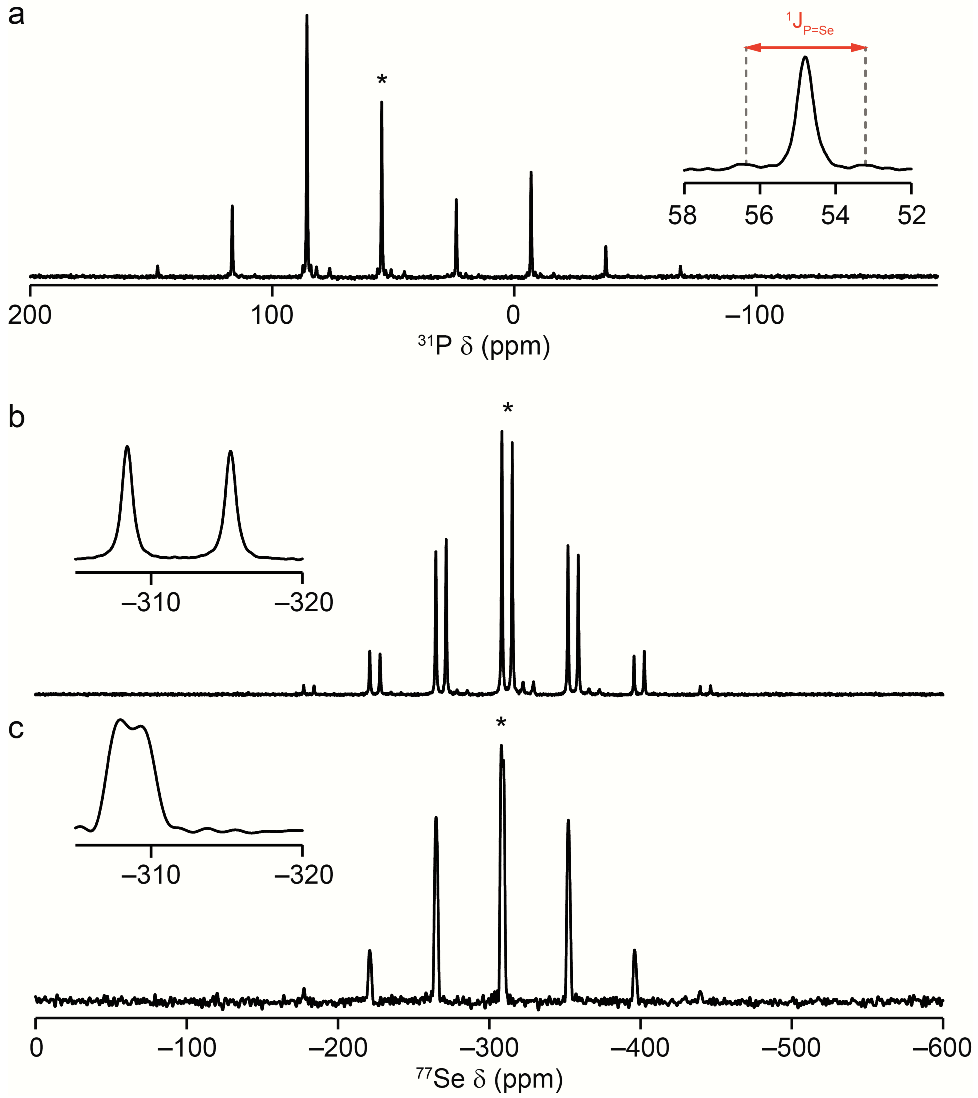

Figure S5.2. (a) ^{31}P (14.1 T, 7.5 kHz) MAS NMR spectra of **4**. (b, c) ^{77}Se (14.1 T, 5 kHz) CP MAS NMR spectra of **4**, acquired (b) without and (c) with ^{31}P decoupling. Isotropic resonances (marked with *) are expanded as insets.

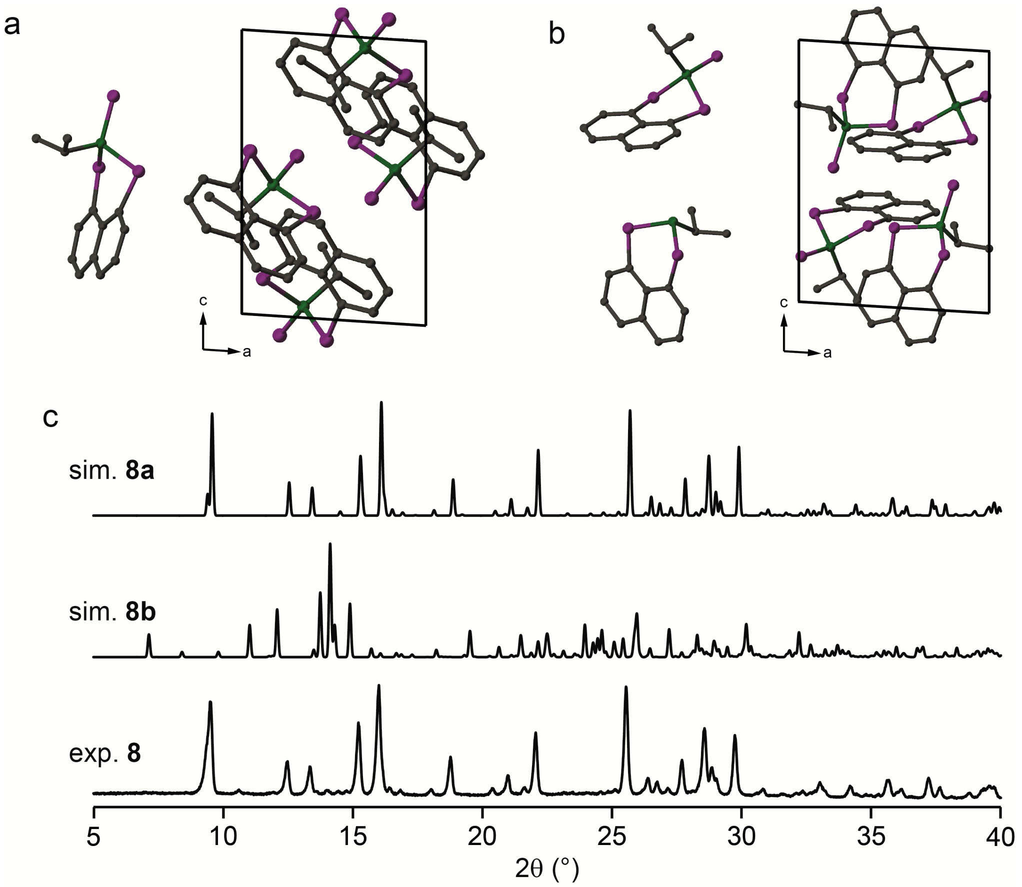

Figure S5.3. (a, b) Asymmetric units (black squares) and packing motifs for (a) **8a** and (b) **8b**. (c) Comparison of the experimental and simulated PXRD patterns for **8**. Atoms are coloured with C = grey, P = green and Se = purple. H atoms are omitted for clarity.

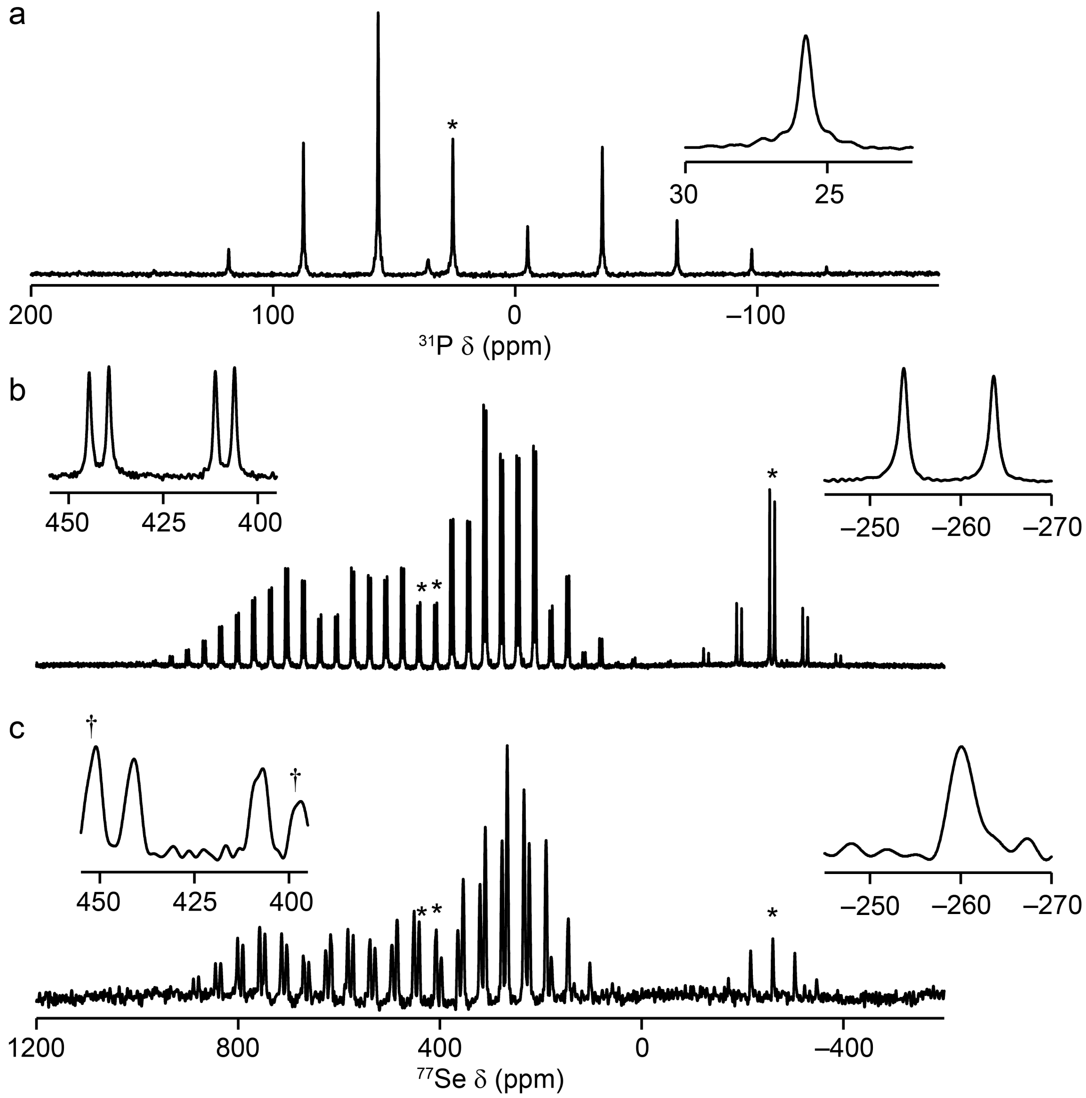

Figure S5.4. (a) ^{31}P (14.1 T, 7.5 kHz) MAS NMR spectra of **8**. (b) ^{77}Se (9.4 T, 5 kHz) and (c) ^{77}Se (14.1 T, 5 kHz) CP MAS NMR spectra of **8**, acquired (b) without and (c) with ^{31}P decoupling. Isotropic resonances (marked with *) are expanded as insets. In (c), spinning sidebands are marked † in the inset.

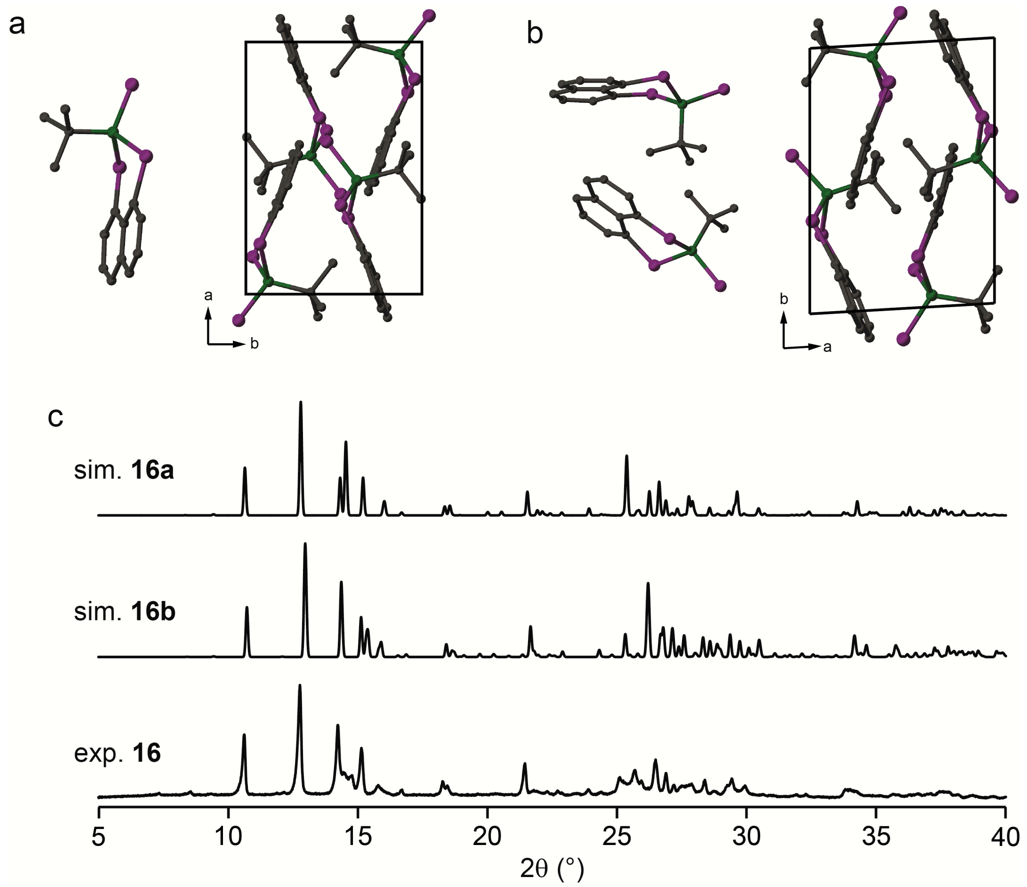

Figure S5.5. (a) Asymmetric unit and packing motif and (b) comparison of the experimental and simulated PXRD patterns for **16**. Atoms are coloured with C = grey, P = green and Se = purple. H atoms are omitted for clarity.

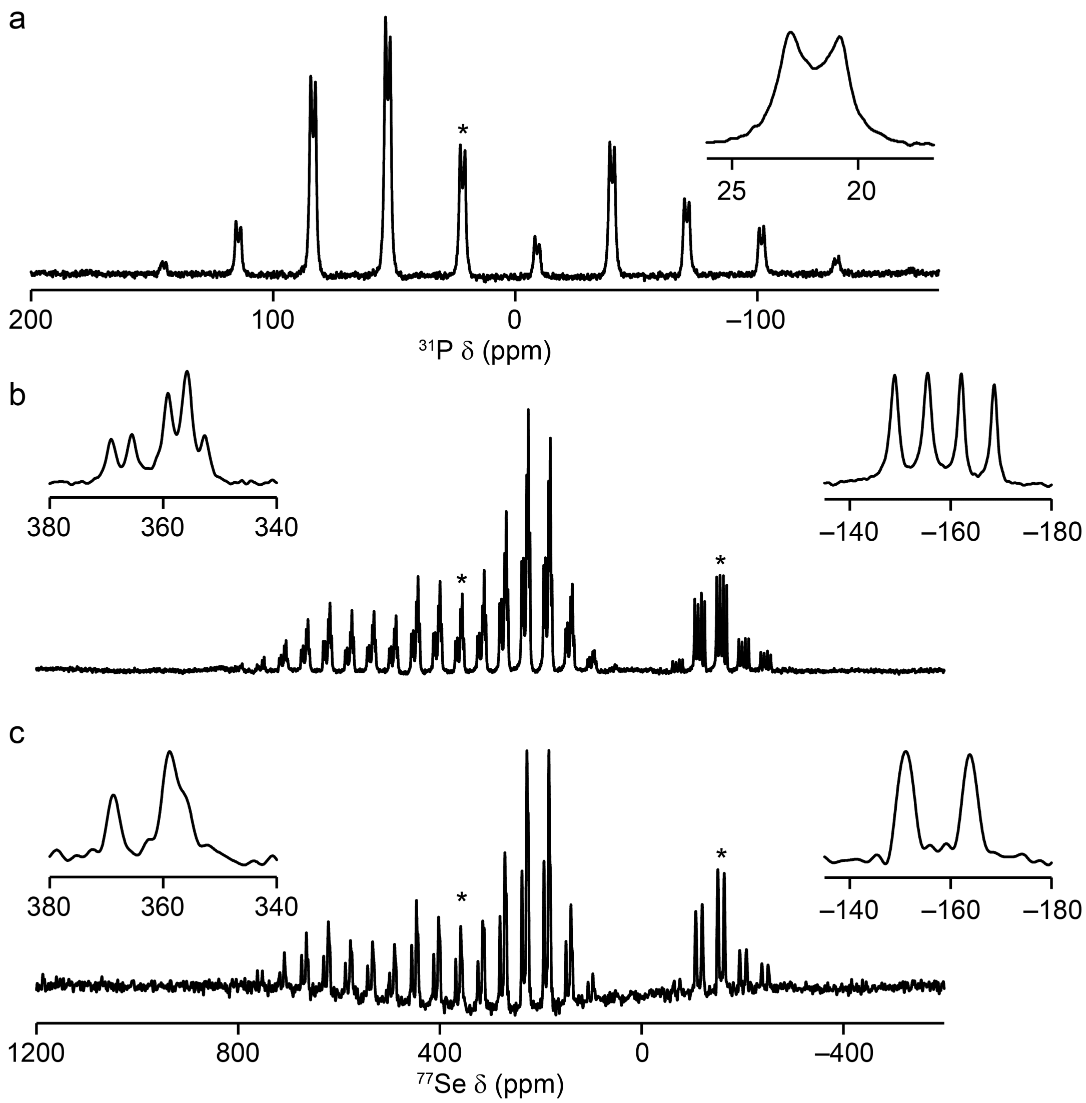

Figure S5.6. (a) ^{31}P (14.1 T, 7.5 kHz) MAS NMR spectrum of **16**. (b, c) ^{77}Se (14.1 T, 5 kHz) CP MAS NMR spectra of **16**, acquired (b) without and (c) with ^{31}P decoupling. Isotropic resonances (marked with *) are expanded as insets.

S6. Experimental shielding tensors

Table S6.1. Experimental ^{31}P NMR parameters (isotropic chemical shift, δ_{iso}^{exp}, span, Ω^{exp}, skew, κ^{exp}, and principal tensor components, δ_{ii}^{exp}, for all compounds.

	**1**	**2**	**3**	**4**	**5**	**6**	**7**	**8**
E, R, X	S, ^{i}Pr, –	S, ^{i}Pr, O	S, ^{i}Pr, S	S, ^{i}Pr, Se	Se, ^{i}Pr	Se, ^{i}Pr, O	Se, ^{i}Pr, S	Se, ^{i}Pr, Se
δ_{iso}^{exp} (ppm)	6<br>4<br>3	36	66<br>64<br>62	55	–2	14	44<br>41	26
Ω^{exp} (ppm)	169<br>184<br>209	303	141<br>177<br>187	159	198	282	207<br>221	202
κ^{exp}	0.9<br>0.4<br>0.3	0.8	0.9<br>0.8<br>0.8	0.9	0.4	1.0	0.9<br>0.7	0.8
δ_{11}^{exp} (ppm)	67<br>85<br>96	148	116<br>128<br>130	111	84	108	117<br>124	101
δ_{22}^{exp} (ppm)	54<br>26<br>24	114	108<br>114<br>114	102	24	108	106<br>95	78
δ_{33}^{exp} (ppm)	–103<br>–99<br>–112	–155	–25<br>–49<br>–57	–48	–114	–174	–90<br>–97	–101
	**9**	**10**	**11**	**12**	**13**	**14**	**15**	**16**
E, R, X	S, ^{t}Bu, –	S, ^{t}Bu, O	S, ^{t}Bu, S	S, ^{t}Bu, Se	Se, ^{t}Bu, –	Se, ^{t}Bu, O	Se, ^{t}Bu, S	Se, ^{t}Bu, Se
δ_{iso}^{exp} (ppm)	–	47	72<br>71	56<br>54	6	30	43	23<br>21
Ω^{exp} (ppm)	–	301	303<br>302	283<br>282	231	320	243	229<br>242
κ^{exp}	–	0.9	0.9<br>0.9	0.9<br>0.9	0.5	0.8	0.9	0.8<br>0.7
δ_{11}^{exp} (ppm)	–	150	179<br>175	156<br>154	103	149	128	105<br>113
δ_{22}^{exp} (ppm)	–	143	161<br>165	137<br>134	44	113	116	87<br>78
δ_{33}^{exp} (ppm)	–	–151	–124<br>–127	–126<br>–127	–128	–171	–115	–124<br>–128

Table S6.2. Experimental ^{77}Se NMR parameters (isotropic chemical shift, δ_{iso}^{exp}, span, Ω^{exp}, skew, κ^{exp}, and principal tensor components, δ_{ii}^{exp}, for all compounds.

	**4**	**5**	**6**	**7**	**8**
E, R, X	S, ^{i}Pr, Se	Se, ^{i}Pr, –	Se, ^{i}Pr, O	Se, ^{i}Pr, S	Se, ^{i}Pr, Se
δ_{iso}^{exp} (ppm)	–309	280	486<br>474	441<br>439<br>432<br>412	442<br>408<br>–260
Ω^{exp} (ppm)	188	589	866<br>906	745<br>703<br>804<br>732	696<br>736<br>197
κ^{exp}	–0.03	–1.0	–0.6<br>–0.4	–0.8<br>–1.0<br>–0.8<br>–0.8	–0.9<br>–1.0<br>–0.9
δ_{11}^{exp} (ppm)	–214	672	1000<br>991	913<br>906<br>939<br>880	893<br>894<br>–133
δ_{22}^{exp} (ppm)	–311	84	327<br>346	245<br>209<br>223<br>207	233<br>171<br>–317
δ_{33}^{exp} (ppm)	–402	83	133<br>85	168<br>203<br>135<br>148	197<br>158<br>–330

	**12**	**13**	**14**	**15**	**16**
E, R, X	S, ^{t}Bu, Se	Se, ^{t}Bu, –	Se, ^{t}Bu, O	Se, ^{t}Bu, S	Se, ^{t}Bu, Se
δ_{iso}^{exp} (ppm)	–46<br>–56	213<br>179	443<br>423	364<br>358	368<br>359<br>356<br>–152<br>–165
Ω^{exp} (ppm)	850<br>979	447<br>470	721<br>782	592<br>560	642<br>628<br>554<br>142<br>124
κ^{exp}	–0.9<br>–0.9	–0.9<br>–1.0	–0.4<br>–0.3	–0.9<br>–1.0	–0.9<br>–0.8<br>–0.9<br>0.4<br>0.9
δ_{11}^{exp} (ppm)	515<br>516	505<br>492	854<br>859	748<br>731	785<br>761<br>713<br>–90<br>–119
δ_{22}^{exp} (ppm)	–318<br>–321	77<br>23	341<br>331	188<br>172	178<br>182<br>196<br>–131<br>–127

δ_{33}^{exp} (ppm)	–335<br>–362	58<br>23	133<br>78	156<br>171	143<br>133<br>159<br>–232<br>–243

S7. Comparison of calculated and experimental J couplings

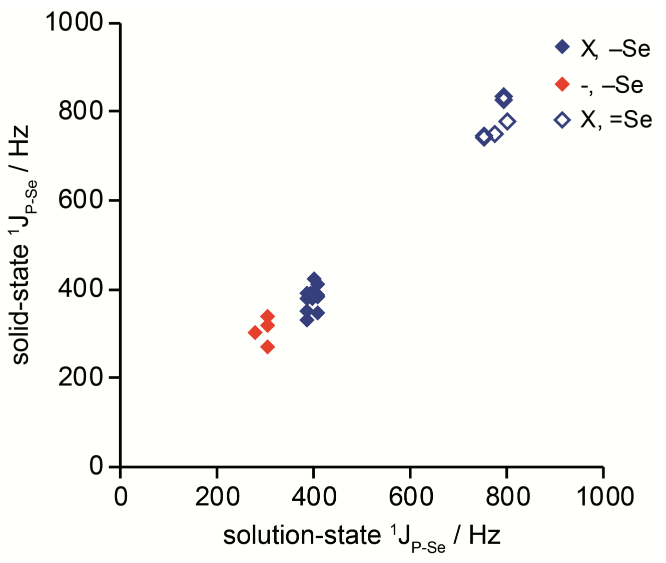

Figure S7.1. Plot of experimental $^1J(^{31}P\text{-}^{77}Se)$ coupling in the solid state against those in solution. The closed points indicate Se = E and the open points indicate Se = X.
